# The Nature of Nonclassical Carbonyl Ligands Explained by Kohn–Sham Molecular Orbital Theory

**DOI:** 10.1002/chem.202003768

**Published:** 2020-11-03

**Authors:** Stephanie C. C. van der Lubbe, Pascal Vermeeren, Célia Fonseca Guerra, F. Matthias Bickelhaupt

**Affiliations:** ^1^ Department of Theoretical Chemistry Amsterdam Institute of, Molecular and Life Sciences (AIMMS) Amsterdam Center of, Multiscale Modeling (ACMM) Vrije Universiteit Amsterdam De Boelelaan 1083 1081 HV Amsterdam The Netherlands; ^2^ Leiden Institute of Chemistry Gorlaeus Laboratories Leiden University Einsteinweg 55 2333 CD Leiden The Netherlands; ^3^ Institute for Molecules and Materials Radboud University Nijmegen Heyendaalseweg 135 6525 AJ Nijmegen The Netherlands

**Keywords:** bonding analysis, carbonyl ligands, density functional calculations, energy decomposition analysis, metal–ligand binding

## Abstract

When carbonyl ligands coordinate to transition metals, their bond distance either increases (classical) or decreases (nonclassical) with respect to the bond length in the isolated CO molecule. C−O expansion can easily be understood by π‐back‐donation, which results in a population of the CO's π*‐antibonding orbital and hence a weakening of its bond. Nonclassical carbonyl ligands are less straightforward to explain, and their nature is still subject of an ongoing debate. In this work, we studied five isoelectronic octahedral complexes, namely Fe(CO)_6_^2+^, Mn(CO)_6_^+^, Cr(CO)_6_, V(CO)_6_^−^ and Ti(CO)_6_^2−^, at the ZORA‐BLYP/TZ2P level of theory to explain this nonclassical behavior in the framework of Kohn–Sham molecular orbital theory. We show that there are two competing forces that affect the C−O bond length, namely electrostatic interactions (favoring C−O contraction) and π‐back‐donation (favoring C−O expansion). It is a balance between those two terms that determines whether the carbonyl is classical or nonclassical. By further decomposing the electrostatic interaction Δ*V*_elstat_ into four fundamental terms, we are able to rationalize why Δ*V*_elstat_ gives rise to the nonclassical behavior, leading to new insights into the driving forces behind C−O contraction.

## Graphical abstract

**Classical or nonclassical?** We show that there are two competing forces that affect the CO bond length in transition metal complexes, namely electrostatic interactions (favoring CO contraction) and π‐back‐donation (favoring CO expansion). The origin is elucidated by quantitative MO‐theory, leading to new insights into the driving forces behind nonclassical behavior.

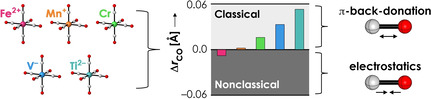

## Introduction

The CO molecule is one of the most important ligands in transition metal chemistry, and has been used across a wide range of chemical disciplines.[^1^, ^2^, ^3^] Due to their electronic flexibility, that is, the ability to both donate electrons to and accept electrons from the transition metal, CO ligands play a prominent role in the design of catalysts that are being used in many applications, such as bond activation, hydroformylation and hydrocarboxylation.[^4^, ^5^, ^6^, ^7^, ^8^] This has led many chemists to study the bonding between M and CO in metal‐carbonyl complexes in more detail.[^9^, ^10^, ^11^, ^12^, ^13^]

**Scheme 1:**
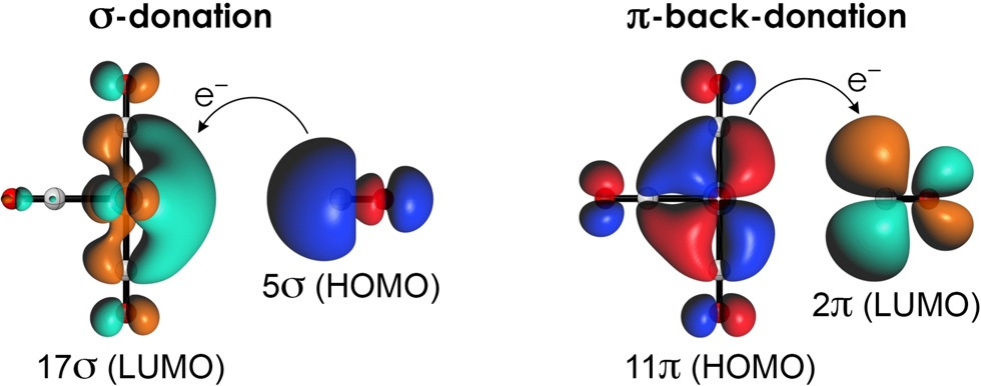
Representation of the orbital interactions between carbonyl ligands and TM(CO)_5_ complexes. Isosurfaces (at 0.03 Bohr^−3/2^) were generated by using the fragment analysis on Cr(CO)_6_ with Cr(CO)_5_ as one fragment and CO as the other fragment, computed at the ZORA‐BLYP/TZ2P level of theory.

When carbonyl coordinates to a transition metal, the C−O bond length can either increase or decrease with respect to the bond length of isolated CO. In the case of bond length expansion, the carbonyl complex is said to be classical, which is usually explained by the Dewar–Chatt–Duncanson (DCD) model (Scheme 1).[^14^, ^15^] In this model, two types of orbital interactions contribute to the formation of the CO‐transition metal (TM) bond, namely the CO → TM σ‐donation and CO ← TM π‐back‐donation. Since π‐back‐donation results in a population of the CO's antibonding π‐orbital, the C−O bond is weakened and thus expanded upon coordination to the metal, which is usually accompanied by a redshift of the C−O stretching frequency *ν*_CO_.

On the other hand, nonclassical carbonyl complexes come with shorter C−O bond lengths and blue‐shifted stretching frequencies *ν*_CO_ with respect to free CO. Nonclassical carbonyls were first defined in 1994 by Strauss and co‐workers for σ‐only metal complexes that cannot participate in π‐back‐bonding,^[16]^ and are usually (but not always)[^17^, ^18^] encountered in cationic complexes.[^19^, ^20^, ^21^, ^22^] In contrast to classical carbonyls, nonclassical carbonyls are less straightforward to explain and have therefore been a subject of ongoing research in which different explanations have been proposed.[^18^, ^22^, ^23^, ^24^, ^25^, ^26^, ^27^, ^28^]

In one explanation, the CO → TM σ‐donation strengthens the C−O bond because the 5σ HOMO is slightly antibonding in nature.[^29^, ^30^, ^31^] This is supported by the observations that: 1) CH_3_CO^+^, HCO^+^ and BH_3_CO^+^ are nonclassical σ‐only systems, suggesting that σ‐donation leads to a strengthening of the C−O bond,^[16]^ and 2) the removal of an electron from the 5σ orbital of CO results in a shortening of the C−O bond and blueshift of its stretching frequency.[^25^, ^32^] However, as the resulting CO^+^ cation has a frequency shift *ν*_CO_ of only 41 cm^−1^, while there are complexes with considerably larger blueshifts than CO^+^ (e.g., blueshifts of around 120 cm^−1^ for [Pt(CO)_4_][Sb_2_F_11_]_2_),^[33]^ Aubke et al. have argued that the antibonding nature of 5σ does not satisfactorily explain the nonclassical behavior and that there are other factors at play instead.^[34]^ Additionally, it has been suggested that the 5σ orbital is actually bonding^[35]^ or (close to) to nonbonding[^24^, ^36^, ^37^] in nature, meaning that depopulation of the 5σ HOMO would either result in C−O expansion or no change of the C−O bond length at all.

Another view is that nonclassical carbonyl ligands are driven by electrostatic and polarization effects.[^18^, ^24^, ^25^, ^35^, ^38^] In isolated CO, the bonding orbitals are polarized towards the oxygen atom, but the introduction of a (partial) positive charge near the carbon atom induces polarization from O to C, resulting in a more covalent and thus a shorter C−O bond. In line with this observation is that a positive charge on the oxygen side of CO results in a weakening, instead of a strengthening of the C−O bond.[^18^, ^25^, ^35^] In a more recent study, Tarantelli and co‐workers argue that the polarization in the π‐electron system is the determining factor for C−O bond length changes.^[22]^ They also show that a variety of classical and nonclassical metal complexes have very similar σ‐charge rearrangements, which suggests that σ‐donation is not a determining factor for geometric changes in CO.

More recently, Head‐Gordon and co‐workers identified the change in dipole moment as the driving force behind C−O contraction.[^27^, ^28^] As the C−O distance decreases, its dipole moment (which has its negative side on C because of the large lobe of MO 5σ on C pointing away from O,^[35]^ see also Scheme 1) becomes larger, which enhances the electrostatic interaction when coordinating to metals with a (partial) positive charge. They also argue that the main effect of the σ‐orbital interaction is the reduction of the C−M bond length, which further enhances the electrostatic interaction that drives C−O contraction.

In the current work, we study five isoelectronic octahedral transition metal complexes with the aim to pinpoint the driving forces behind nonclassical carbonyl ligands. Our results show that there are two determining factors, namely the electrostatic interaction (favoring C−O contraction) and π‐back‐donation (favoring C−O expansion). It is an interplay between these two terms that determines whether the CO will be classical or nonclassical in nature. By further decomposing the electrostatic interaction Δ*V*_elstat_ into four fundamental terms, we have been able to trace the origin of the differences in Δ*V*_elstat_, leading to new insights into the driving forces behind C−O contraction.

## Results and Discussion

**Figure 1:**
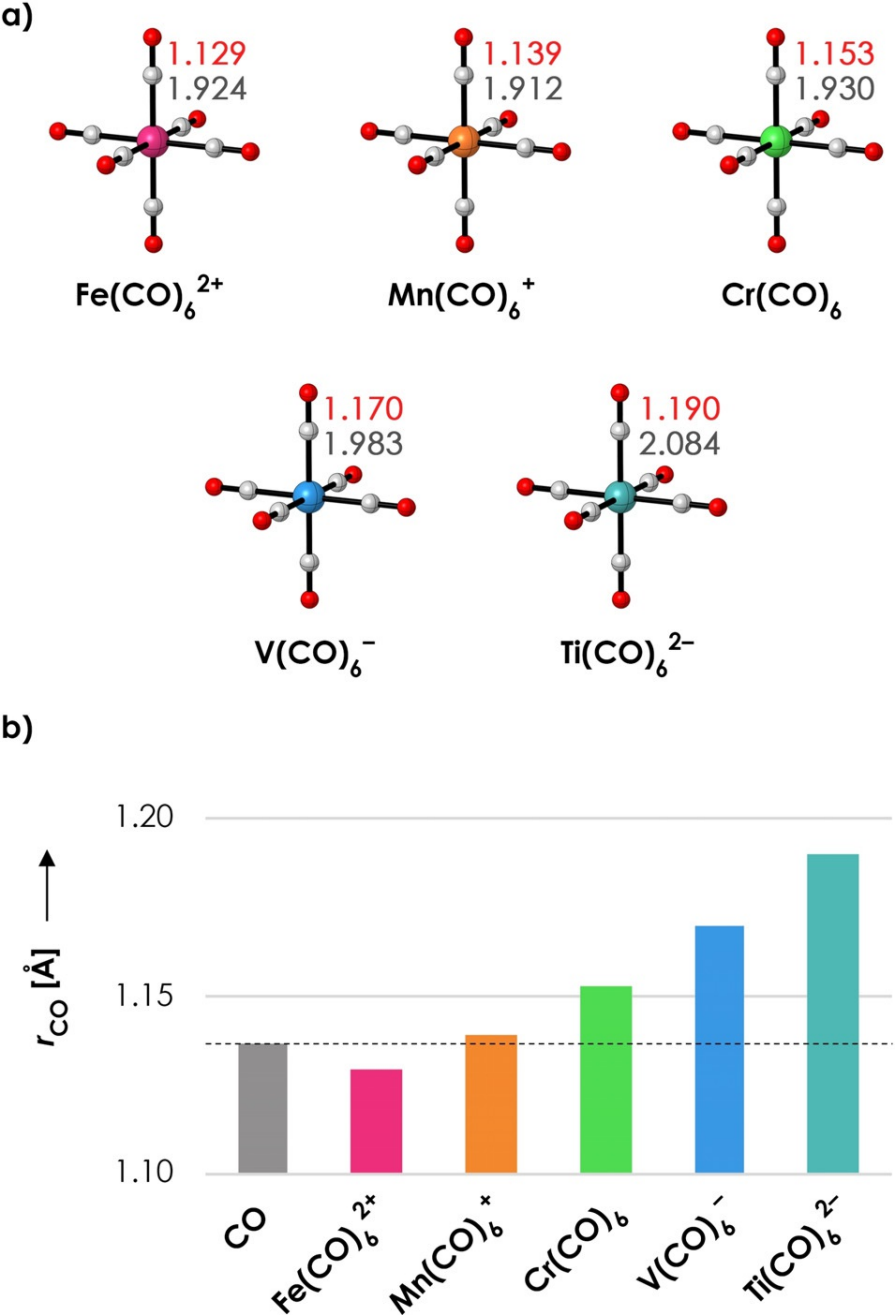
a) Studied molecular systems with C−O (red) and C−M (gray) distances (in Å). b) C−O distances (in Å). The dashed line represents the C−O distance of isolated CO. All data obtained at the ZORA‐BLYP/TZ2P level of theory.

We studied five octahedral systems, namely Fe(CO)_6_^2+^, Mn(CO)_6_^+^, Cr(CO)_6_, V(CO)_6_^−^ and Ti(CO)_6_^2−^. The advantage of these systems is that they are isoelectronic, which will help us to pinpoint the driving forces behind nonclassical behavior. As can be seen in Figure 1 a, the C−O bond length is the shortest in Fe(CO)_6_^2+^ (1.129 Å) and gradually expands as the charge goes from +2 to −2, reaching a maximum in Ti(CO)_6_^2−^ (1.190 Å). Here, we note that all properties studied in this paper change in the same orderly manner when going from Fe(CO)_6_^2+^ to Ti(CO)_6_^2−^. Hence, we will often restrict our discussion to the two extremes, that is, Fe(CO)_6_^2+^ and Ti(CO)_6_^2−^. Comparing the C−O bond lengths in the transition metal complexes with the bond length of 1.137 Å in isolated CO (Figure 1 b), we see that Fe(CO)_6_^2+^ is the only system with contracted CO ligands, and hence the only nonclassical system. The C−O bond lengths in the other four systems are all expanded with respect to isolated CO, with the largest C−O expansion in Ti(CO)_6_^2−^.

**Scheme 2:**
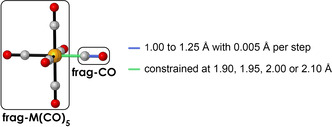
Schematic representation of the fragment‐based approach used in this work. The frag‐M(CO)_5_ is always kept in the same geometry as in the fully optimized overall system.

To understand the driving forces behind C−O contraction and expansion upon the formation of an octahedral complex, we employed the energy decomposition analysis (EDA) as a function of the C−O bond length.[^39^, ^40^] In this fragment‐based approach, the interaction energy Δ*E*_int_ is decomposed into three physically meaningful terms, namely the electrostatic interaction Δ*V*_elstat_, Pauli repulsion Δ*E*_Pauli_ and the orbital interaction Δ*E*_oi_ (see Theoretical Methods for a theoretical overview). We took one CO ligand as one fragment (frag‐CO), and the rest of the molecular system as the other fragment (frag‐M(CO)_5_). The C−O distance of frag‐CO was increased in a stepwise manner from 1.00 to 1.25 Å with 0.005 Å per step, resulting in 51 steps in total. The rest of the system, frag‐M(CO)_5_, was always frozen in the same geometry as acquired from the fully optimized overall system M(CO)_6_. To eliminate any effects originating from the differences in M–CO distance, we also constrained the distance between the carbon atom on frag‐CO and the metal atom in frag‐M(CO)_5_ at 1.90, 1.95, 2.00 and 2.10 Å. A schematic representation of our fragment‐based approach is given in Scheme 2. In this study, we will limit our discussion to the results that were obtained at a C−M distance of 1.95 Å because this distance is the closest to the average C−M bond length of the five studied systems. The other C−M distances gave identical trends and are provided in Figures S1–3.

**Figure 2:**
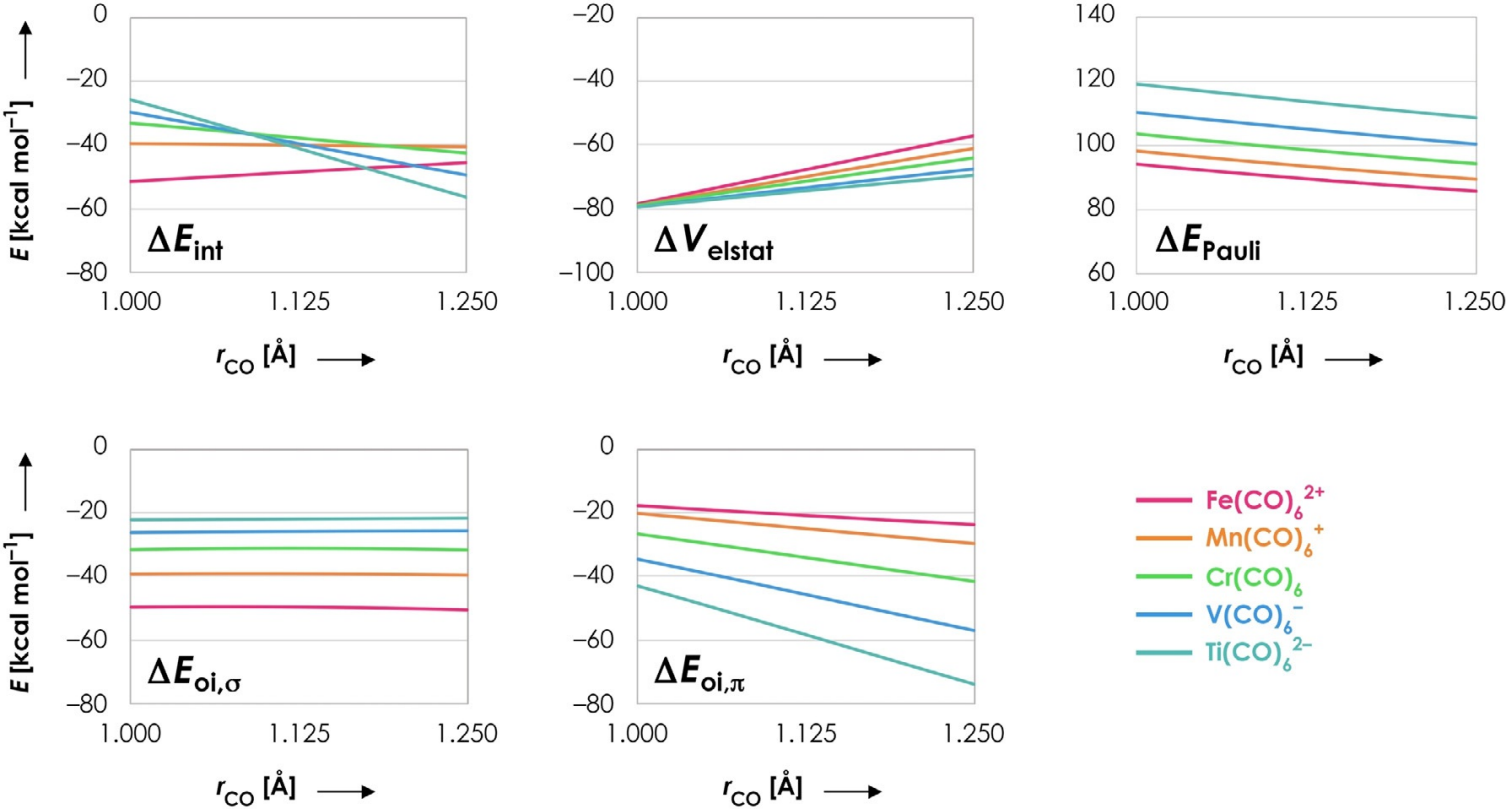
Energy decomposition terms (in kcal mol^−1^) as a function of the C−O distance *r* (in Å) for Fe(CO)_6_^2+^ (pink), Mn(CO)_6_^+^ (orange), Cr(CO)_6_ (green), V(CO)_6_^−^ (blue) and Ti(CO)_6_^2−^ (turquoise). One C−O distance (frag‐CO) has been varied in a stepwise manner from 1.00 to 1.25 Å while keeping its corresponding M‐C distance fixed at 1.95 Å; the rest of the system (frag‐M(CO)_5_) is frozen in the same geometry as the fully optimized overall system. All data obtained at the ZORA‐BLYP/TZ2P level of theory.

As can be seen in Figure 2, the interaction energy Δ*E*_int_ between CO and Fe(CO)_5_^2+^ becomes more stable when CO is contracted, which is in line with its nonclassical nature. For the other complexes, decreasing the C−O distance becomes increasingly unfavorable when going from Mn(CO)_6_^+^ to Ti(CO)_6_^2−^. This is dictated by the interplay of two terms, namely the electrostatic interaction Δ*V*_elstat_ and π‐back‐donation Δ*E*_oi,π_. The electrostatic interaction favors C−O contraction for all molecular systems, but the tendency for C−O contraction is the strongest for Fe(CO)_6_^2+^ (largest slope) and smallest for Ti(CO)_6_^2−^ (smallest slope). The π‐back‐donation favors C−O expansion for all five systems, but the tendency for C−O expansion is the strongest for Ti(CO)_6_^2−^ (largest slope) and smallest for Fe(CO)_6_^2+^ (smallest slope). Hence, the blue‐shifting behavior of Fe(CO)_5_^2+^ is mainly dictated by Δ*V*_elstat_, whereas the red‐shifting behavior of Ti(CO)_5_^2−^ is mainly dictated by Δ*E*_oi,π_. The Pauli repulsion Δ*E*_Pauli_ favors C−O expansion in all five systems, which has to be overcome by the electrostatic interaction Δ*V*_elstat_ to obtain nonclassical behavior. The reason for this tendency is that C−O contraction enlarges the amplitude of MO 5σ on C, resulting in a larger overlap with the filled orbitals on frag‐M(CO)_5_ and therefore a larger Pauli repulsion (see below and Discussion 1 in the Supporting Information). Nevertheless, the slope of Δ*E*_Pauli_, and thus its tendency for C−O expansion, is the same for all five molecular systems, meaning that the Pauli repulsion does not explain the differences between Fe(CO)_6_^2+^ and Ti(CO)_6_^2−^. Finally, the σ‐orbital interaction Δ*E*_oi,σ_ is approximately constant along the whole C−O bond length, which is in line with the close to nonbonding nature of the 5σ orbital.[^24^, ^36^, ^37^] Hence, the σ‐orbital interaction does not dictate whether the molecular system will be classical or nonclassical in nature.

**Figure 3:**
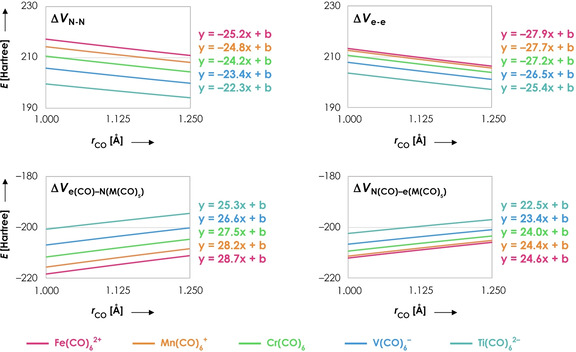
Decomposition of electrostatic interaction (in Hartree) as a function of the C−O distance *r* (in Å) for Fe(CO)_6_^2+^ (pink), Mn(CO)_6_^+^ (orange), Cr(CO)_6_ (green), V(CO)_6_^−^ (blue) and Ti(CO)_6_^2−^ (turquoise). One C−O distance (frag‐CO) has been varied in a stepwise manner from 1.00 to 1.25 Å while keeping its corresponding M‐C distance fixed at 1.95 Å; the rest of the system (frag‐M(CO)_5_) is frozen in the same geometry as the fully optimized overall system. Linear equations are given to see the differences in slope (*R* ^2^=1.00 for each linear regression). All data obtained at the ZORA‐BLYP/TZ2P level of theory.

To understand why the electrostatic interaction has a varying tendency for C−O contraction when going from Fe(CO)_6_^2+^ to Ti(CO)_6_^2−^, we further decomposed Δ*V*_elstat_ into the following terms [Eq. 1]:

(1) $$\Delta V_{\mathrm{elstat}}\;\;=\;\sum_{\begin{array}{c} \alpha \epsilon A\\ \beta \epsilon B \end{array}}\frac{Z_{\alpha }Z_{\beta }}{R_{\alpha \beta }}-\int \sum_{\alpha \epsilon A}\frac{Z_{\alpha }\rho_{B}\left(r\right)}{\left|r-R_{\alpha }\right|}\mathrm{dr}-\int \sum_{\beta \epsilon B}\frac{Z_{\beta }\rho_{A}\left(r\right)}{\left|r-R_{\beta }\right|}\mathrm{dr}\;+\int \int \frac{\rho_{A}\left(r_{1}\right)\rho_{B}\left(r_{2}\right)}{r_{12}}dr_{1}dr_{2}=\;\Delta V_{N-N}\;+\;\Delta V_{N-e}\;+\;\Delta V_{e-N}\;+\;\Delta V_{e-e}$$

where A and B refer to frag‐CO and frag‐M(CO)_5_, respectively. The first term is the repulsive Coulombic interaction between the nuclei in frag‐CO with those in frag‐M(CO)_5_, the second and third terms are the attractive Coulombic interactions between the nuclei of frag‐CO with the electrons in frag‐M(CO)_5_ and vice versa, and the final term is the repulsive Coulombic interaction between the electrons in frag‐CO with the electrons in frag‐M(CO)_5_. The results of this decomposition are shown in Figure 3.

Both the nucleus–nucleus repulsion Δ*V*_N‐N_ and electron–electron repulsion Δ*V*_e‐e_ favor C−O expansion, while the electron–nuclei terms Δ*V*_N‐e_ and Δ*V*_e‐N_ favor C−O contraction. Interestingly, the tendency of Δ*V*_N‐N_ and Δ*V*_e‐e_ to favor C−O *expansion* is the strongest for Fe(CO)_6_^2+^ (largest negative slope), yet the tendency of the total electrostatic interaction to induce C−O *contraction* is also the largest for Fe(CO)_6_^2+^. Hence, both repulsive terms Δ*V*_N‐N_ and Δ*V*_e‐e_ do not explain the relatively large tendency for C−O contraction in Fe(CO)_6_^2+^. The attractive terms Δ*V*_N‐e_ and Δ*V*_e‐N_ have the largest slope for Fe(CO)_6_^2+^ and smallest slope for Ti(CO)_6_^2−^. Thus, the electrostatic interaction has the largest propensity for C−O contraction in Fe(CO)_6_^2+^ because of the relatively fast stabilization of the interaction between: 1) the electrons in CO with the nuclei in frag‐M(CO)_5_ (Δ*V*_e‐N_), and 2) the nuclei in CO with the electrons in frag‐M(CO)_5_ (Δ*V*_N‐e_) upon C−O contraction. We will now further rationalize these observations.

**Figure 4:**
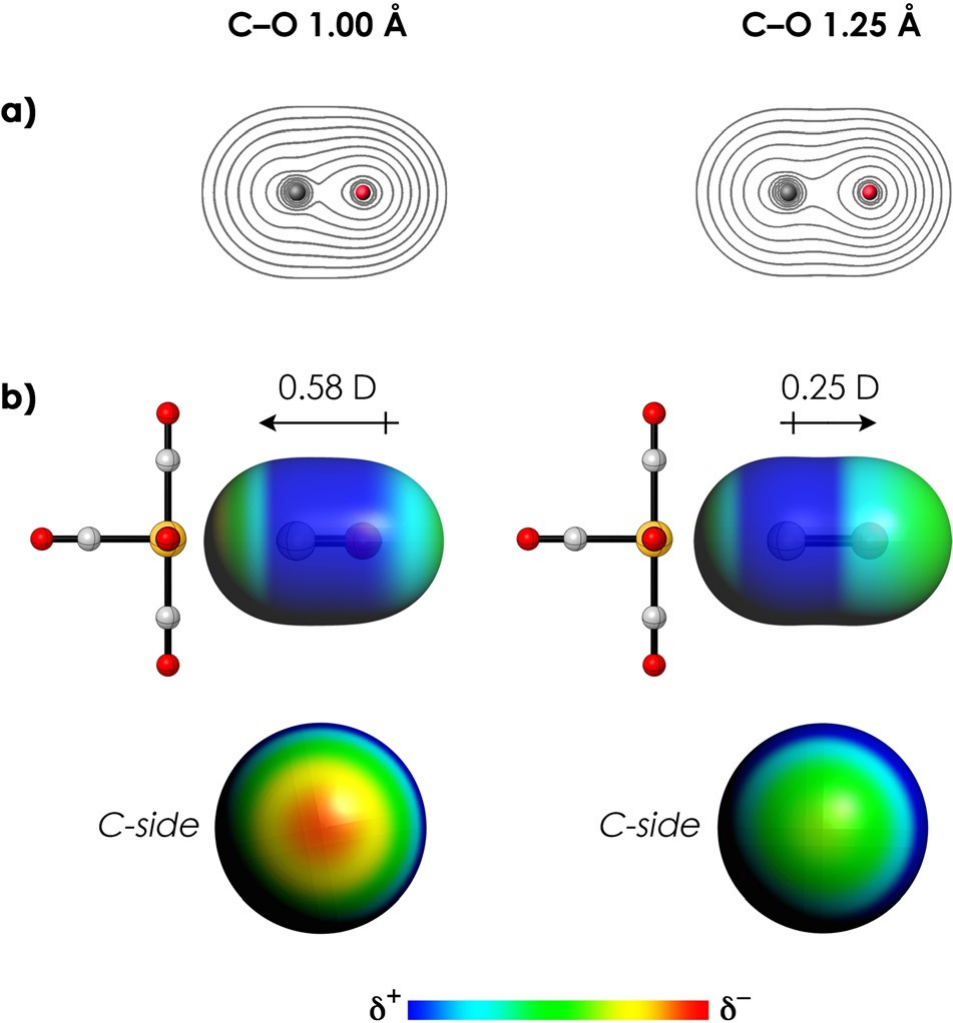
Electronic density analysis of isolated CO with *r*=1.00 Å (left) and *r*=1.25 Å (right). a) Density contours from 0.01 to 5.0 Bohr^−3^ on a logarithmic scale. The maxima are located on the positions of the nuclei. b) Electrostatic potential surfaces (at 0.01 Bohr^−3^) from −0.05 (red) to 0.05 (blue) Hartree e^−1^ and dipole moments (in Debye) for isolated CO. The C atom becomes more negative upon C−O contraction. All data obtained at the ZORA‐BLYP/TZ2P level of theory.

We start with the electrostatic interaction Δ*V*_e‐N_ of the electrons in CO with the nuclei in frag‐M(CO)_5_. The first question is more general and applies to all five molecular systems; why does Δ*V*_e‐N_ become more stable when the C−O distance is decreased? We know from Equation (1) that Δ*V*_e‐N_ is determined by four factors, namely the charge and position of the nuclei, and the number and position of the electrons. Three of these factors remain constant when the C−O bond length is varied, namely the nuclear charges, the position of the nuclei (because frag‐M(CO)_5_ is always frozen in one geometry) and the number of electrons. This means that the stabilization of the Δ*V*_e‐N_ can only be caused by the change in the position of the electrons in CO. The position of the electrons upon C−O contraction is changed for two reasons. The first reason (from now on called Effect 1) is that the electron density exhibits maxima at the positions of the nuclei, so decreasing the C−O distance (and thus the distance between O and frag‐M(CO)_5_) automatically decreases the distance between the electrons in CO and the nuclei of frag‐M(CO)_5_ as well (Figure 4 a). The second reason (from now on called Effect 2) is that decreasing the C−O distance leads to a shift of electronic density in the direction of the TM atom, which again decreases the distance between the electrons in CO and nuclei in frag‐M(CO)_5_. This electronic density shift is not only revealed by the electrostatic potential surfaces, but also by the molecular dipole moment of CO, which goes from 0.25 D with its positive side on C to 0.58 D with its positive side on O (Figure 4 b). The latter is in line with recent work by Head‐Gordon et al., who identified the change in dipole moment as one of the drivers behind C−O contraction.[^27^, ^28^] The main reason for this density shift is that C−O contraction increases the antibonding overlap between the 2s orbital on C with the 2p_z_ orbital on O in the 5σ HOMO,^[37]^ resulting in a larger lobe on the carbon atom pointing away from the oxygen atom (Figure S4).

**Figure 5:**
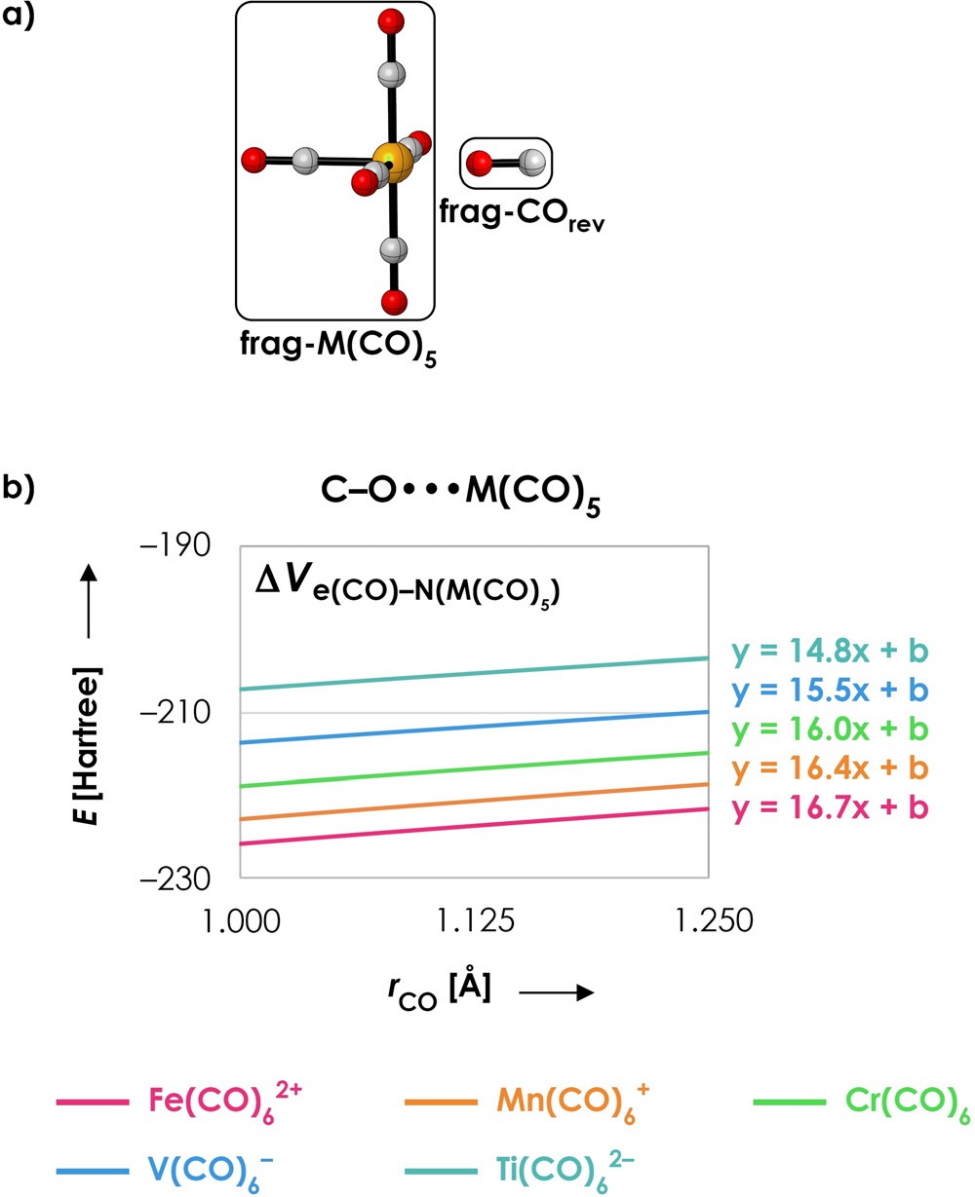
a) Molecular structure of reversed frag‐CO (frag‐CO_rev_) bonded to frag‐M(CO)_5_. b) Electrostatic energy between the electrons in frag‐CO_rev_ with the nuclei in frag‐M(CO)_5_ (in Hartree) as a function of the C−O distance *r* (in Å) for M=Fe^2+^ (pink), Mn^+^ (orange), Cr (green), V^−^ (blue) and Ti^2−^ (turquoise). The C−O distance of frag‐CO_rev_ has been varied in a stepwise manner from 1.00 to 1.25 Å while keeping the corresponding M‐O distance fixed at 1.95 Å; the rest of the system (frag‐M(CO)_5_) is frozen in the same geometry as the fully optimized overall system with all carbonyls coordinating with C. Linear equations show the differences in slopes (*R* ^2^=1.00 for each linear regression). All data obtained at the ZORA‐BLYP/TZ2P level of theory.

To probe the importance of both effects, we computed the Δ*V*_e‐N_ with frag‐CO reversed, which bonds to frag‐M(CO)_5_ with the oxygen atom instead of the carbon atom (Figure 5 a). With CO reversed, Effect 1 will still result in a decreasing e‐N distance upon C−O contraction because the maxima of the electronic density are still located on the nuclei. However, Effect 2 increases, instead of decreases, the e‐N distance upon C−O contraction, because the shift of electronic density from O to C is now away from frag‐M(CO)_5_. If both counteracting effects would be equally important, the Δ*V*_e‐N_ curves as a function of *r*_CO_ would become approximately flat (i.e., zero slope) when CO is reversed. However, as can be seen in Figure 5 b, Δ*V*_e‐N_ still has a tendency for C−O contraction when CO is reversed. This tendency is less pronounced (less steep slopes) in comparison with the normal bonding situation in which frag‐CO coordinates with C (Figure 3), but still substantial. We can therefore conclude that Effect 1 (i.e., the change of the electron‐nucleus distance because the electronic density has its maxima on the positions of the nuclei) is the most important reason for Δ*V*_e‐N_ to have a tendency for C−O contraction.

Next, we address the question of why the stabilization of Δ*V*_e‐N_ upon C−O contraction is the strongest for Fe(CO)_6_^2+^ (largest slope) and the weakest for Ti(CO)_6_^2−^ (smallest slope). In this case, the change in the electronic density of CO is of course the same for all molecular systems, meaning that this cannot explain the different tendencies for C−O contraction. There are instead two other factors that contribute to the difference, namely the positions and the charge of the nuclei. The positions of the nuclei are varying among the different systems because frag‐M(CO)_5_ is frozen in the position of the fully optimized overall system. Hence, Fe^2+^(CO)_5_ has the shortest C−O and C−M distances, while Ti^2−^(CO)_5_ has the largest C−O and C−M distances (Figure 1). To measure the importance of this effect, we redid the computations while freezing Fe(CO)_6_^2+^ and Ti(CO)_6_^2−^ in each other's geometry. As can be seen in Figure S5, the difference in slope between Fe(CO)_6_^2+^ and Ti(CO)_6_^2−^ goes from 3.4 to 2.1 when both systems share the same geometry. Hence, the larger tendency for C−O contraction of Fe(CO)_6_^2+^ is partly caused by the shorter distance between the electrons in CO and nuclei in frag‐M(CO)_5_, which follows from its shorter C−O and C−M distances. However, the main reason for the different tendencies for C−O contraction is the difference in nuclear charge *Z*, which is the lowest for Ti (*Z*=22) and the highest for Fe (*Z*=26). The high nuclear charge of Fe results in a larger number in the numerator [Eq. (1)], and explains why the density change in CO has a more favorable effect when M=Fe than when M=Ti.

The second term that dictates the tendency for C−O contraction is the electrostatic interaction between the nuclei of CO with the electrons in the rest of the complex, Δ*V*_N‐e_. We start again with the more general question of why Δ*V*_N‐e_ becomes more stabilizing for all five systems when the C−O distance is decreased. The number and position of the electrons in frag‐M(CO)_5_ and the nuclear charge of frag‐CO all remain constant upon C−O contraction. Hence, the enhanced electrostatic interaction Δ*V*_N‐e_ can only be caused by the positions of the nuclei in CO. As we decrease the C−O distance, the distance between the nuclei in CO and electrons in frag‐M(CO)_5_ automatically decreases as well, which fully explains the general tendency of Δ*V*_N‐e_ for C−O contraction.

**Figure 6:**
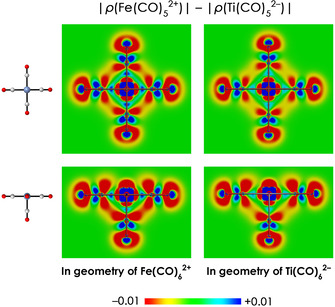
Difference in electronic density distribution between Fe(CO)_5_^2+^ and Ti(CO)_5_^2−^, obtained by subtracting the absolute density of Ti(CO)_5_^2−^ from the absolute density of Fe(CO)_5_^2+^ while both systems share the same geometry (i.e. the geometry of Fe(CO)_6_^2+^ (left) and Ti(CO)_6_^2−^ (right)). Hence, positive values (blue color) correspond to regions where Fe(CO)_5_^2+^ has more electronic density, while negative values (red color) correspond to regions where Ti(CO)_5_^2−^ has more electronic density. Top: viewed from the missing CO (frag‐CO) side. Bottom: viewed from CO in M(CO)_5_. Data obtained at the ZORA‐BLYP/TZ2P level of theory.

Next, we address the question of why this tendency is the strongest for Fe(CO)_6_^2+^ and smallest for Ti(CO)_6_^2−^. Since the position of the oxygen atom is changed in the same way for all molecular systems, the positions of the nuclei cannot be the reason for the different tendencies. Furthermore, the nuclear charge of CO and the number of electrons in frag‐M(CO)_5_ is the same for all molecular systems, the latter because our systems are isoelectronic. Hence, there is only one possibility remaining, which is the position of the electrons. Apparently, the distance between the electrons in frag‐M(CO)_5_ and the nuclei in CO is the smallest in Fe(CO)_6_^2+^, resulting in a smaller denominator and hence the largest response to C−O contraction. The reason for this is that the density in Fe(CO)_5_^2+^ is more localized around the metal atom, while it is more delocalized over the CO ligands in Ti(CO)_5_^2−^. We have visualized this by subtracting the absolute density of Ti(CO)_5_^2−^ from the absolute density of Fe(CO)_5_^2+^ while both systems share the same geometry. As can be seen in Figure 6, Fe(CO)_5_^2+^ has more electronic density around the metal atom (blue color), while Ti(CO)_5_^2−^ has more electronic density away from the central metal (red color). Hence, we can clearly see that the electronic density is more compact in Fe(CO)_5_^2+^ than in Ti(CO)_5_^2−^. This can easily be understood from the higher nuclear charge of Fe, resulting in a larger net attraction of the electrons to the metal and less π back‐donation to the CO ligands (see below), resulting in a more compact electronic density around the metal center in Fe^2+^(CO)_5_.

Summarizing our findings up to here, the electrostatic interaction has the strongest tendency for C−O contraction in Fe(CO)_6_^2+^ and lowest tendency for C−O contraction in Ti(CO)_6_^2−^ for two reasons, namely the attractive interactions between: 1) the electrons in CO and nuclei in frag‐M(CO)_5_, Δ*V*_e‐N_, and 2) the nuclei in CO with the electrons in frag‐M(CO)_5_, Δ*V*_N‐e_. Contracting the C−O distance decreases the distance between the electrons in CO with the nuclei in frag‐M(CO)_5_, resulting in a more stable Δ*V*_e‐N_. As Fe has shorter C−O and C−M bonds and, more importantly, the highest nuclear charge, the stabilization of Δ*V*_e‐N_ is the most important for Fe(CO)_6_^2+^. Contracting the C−O distance also decreases the distance between the nuclei in CO with the electrons in frag‐M(CO)_5_. As Fe has the most compact density, which follows from its higher nuclear charge, this effect is again the most important for Fe(CO)_6_^2+^.

**Figure 7:**
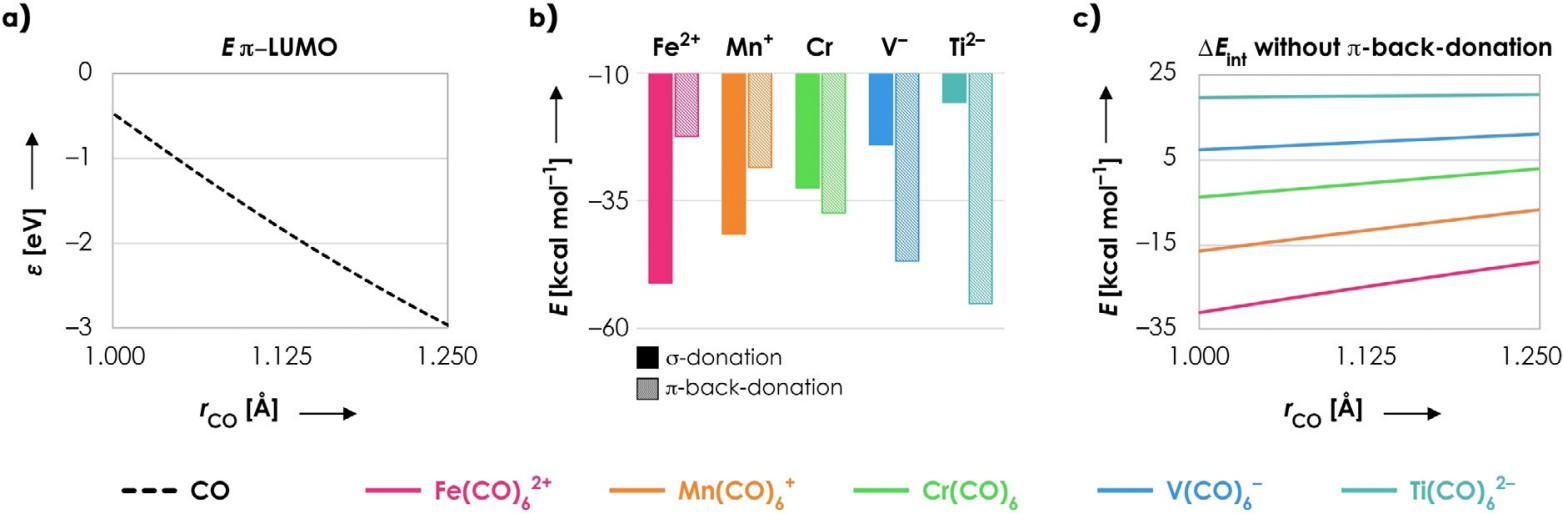
a) π‐LUMO orbital energy of CO as a function of the C−O distance *r* (in Å). b) σ‐ (solid) and π‐ (striped) orbital interactions (in kcal mol^−1^) for the systems at equilibrium. c) Interaction energy Δ*E*_int_ (in kcal mol^−1^) when π‐back‐donation is inhibited (C−M distance fixed at 1.95 Å). All data obtained at the ZORA‐BLYP/TZ2P level of theory.

The next term that will be analyzed in more detail is the π‐back‐donation Δ*E*_oi,π_, which has the strongest tendency for C−O expansion in Ti^2−^(CO)_6_ and smallest tendency for C−O expansion in Fe(CO)_6_^2+^ (Figure 2). Generally, the magnitude of these orbital interactions is proportional to Equation 2:^[41]^

(2) $$\Delta E_{\mathrm{oi}}\propto -\frac{S_{\mathrm{occ},\mathrm{virt}}^{2}}{\left|\epsilon_{\mathrm{occ}}-\epsilon_{\mathrm{virt}}\right|}$$

where *S* is the orbital overlap and *ϵ* is the orbital energy. The orbital interactions can thus be enhanced by a better orbital overlap and a smaller orbital energy gap. We start again with the more general question that applies to all five complexes; why does the π‐back‐donation become weaker upon C−O contraction? As can be seen in Figure 7 a, the π‐LUMO energy goes up in energy when the C−O distance is decreased, which happens because of the increased antibonding overlap between the 2p atomic orbitals on C and O (Figure S6). As the energy of the HOMO on frag‐M(CO)_5_ remains constant upon C−O contraction (because frag‐M(CO)_5_ is kept frozen in the geometry of the fully optimized overall system), the increasing π‐LUMO energy leads to an increase of the π‐HOMO–LUMO gap, and hence a weakening of the orbital interaction Δ*E*_oi,π_ when CO is contracted. On the other hand, the π‐HOMO–LUMO overlap increases when the C−O distance is decreased (Figure S7), which is caused by the larger amplitude of the π‐LUMO on C at shorter C−O distance (Figure S8). As this should lead to a strengthening, instead of a weakening of the orbital interaction, the tendency of Δ*E*_oi,π_ for C−O expansion is thus completely determined by the increase in π‐LUMO energy upon C−O contraction.

The reason that the tendency of Δ*E*_oi,π_ for C−O expansion is the largest for Ti(CO)_6_^2−^ (largest negative slope Figure 2) and smallest for Fe(CO)_6_^2+^ (smallest negative slope Figure 2) is because there is more π‐back‐donation in Ti(CO)_6_^2−^ than in Fe(CO)_6_^2+^. This becomes evident from Figure 7 b, where the charge transfer interactions are given for all systems in equilibrium geometry (the systems with constrained geometries gave identical trends, see Figure S9). Since Ti(CO)_6_^2−^ has the largest and Fe(CO)_6_^2+^ the smallest amount of π‐back‐donation, the effect of the decrease in π‐LUMO energy upon C−O expansion is the most pronounced in Ti(CO)_6_^2−^ and least pronounced in Fe(CO)_6_^2+^. The reason for these differences in Δ*E*_oi,π_ is the energy of the π‐HOMO on frag‐M(CO)_5_, which is the lowest for Fe(CO)_5_^2+^ and highest for Ti(CO)_5_^2−^ (Figure S10). As a result, the π‐HOMO–LUMO gap is the largest for Fe(CO)_6_^2+^ and smallest for Ti(CO)_6_^2−^, which explains why Fe(CO)_6_^2+^ has the smallest amount of π‐back‐donation and Ti(CO)_6_^2−^ has the largest amount of π‐back‐donation. This is not only in line with previous observations,[^10^, ^19^] but can also be understood from simple physical chemistry principles; the positive charge on the Fe^2+^ atom makes it more prone to accept electron density from CO (more σ‐donation), while the negative charge on the Ti^2−^ atom makes it more prone to donate electron density to CO (more π‐back‐donation). In summary, it is the larger amount of π‐back‐donation in Ti(CO)_6_^2−^ that makes the effect of the LUMO destabilization upon C−O contraction more pronounced, which results in the larger tendency for C−O expansion for Ti(CO)_6_^2−^ than for Fe(CO)_6_^2+^.

There is one more question that remains unanswered; how important is π‐back‐donation for the nonclassical behavior in carbonyl ligands? After all, π‐back‐donation has a tendency for C−O expansion, so does it actually play a role in C−O contraction? This question can be answered by recomputing the interaction energy of each complex without any π‐back‐donation by removing all π virtual orbitals. As can be seen in Figure 7 c, Fe(CO)_6_^2+^, Mn(CO)_6_^+^, and Cr(CO)_6_ become nonclassical in nature when π‐back‐donation is inhibited. The interaction energy Δ*E*_int_ becomes positive, i.e., destabilizing, for V(CO)_6_^−^ and Ti(CO)_6_^2−^ when π‐back‐donation is inhibited, showing that π‐back‐donation is necessary for these systems to accomplish a net stabilizing coordination bond. Nevertheless, the slope becomes positive for all systems studied, which means that they all have a tendency for C−O contraction without the presence of π‐back‐donation. Hence, even though π‐back‐donation always favors C−O expansion, its magnitude determines whether the coordination complex will be classical or nonclassical.

**Figure 8:**
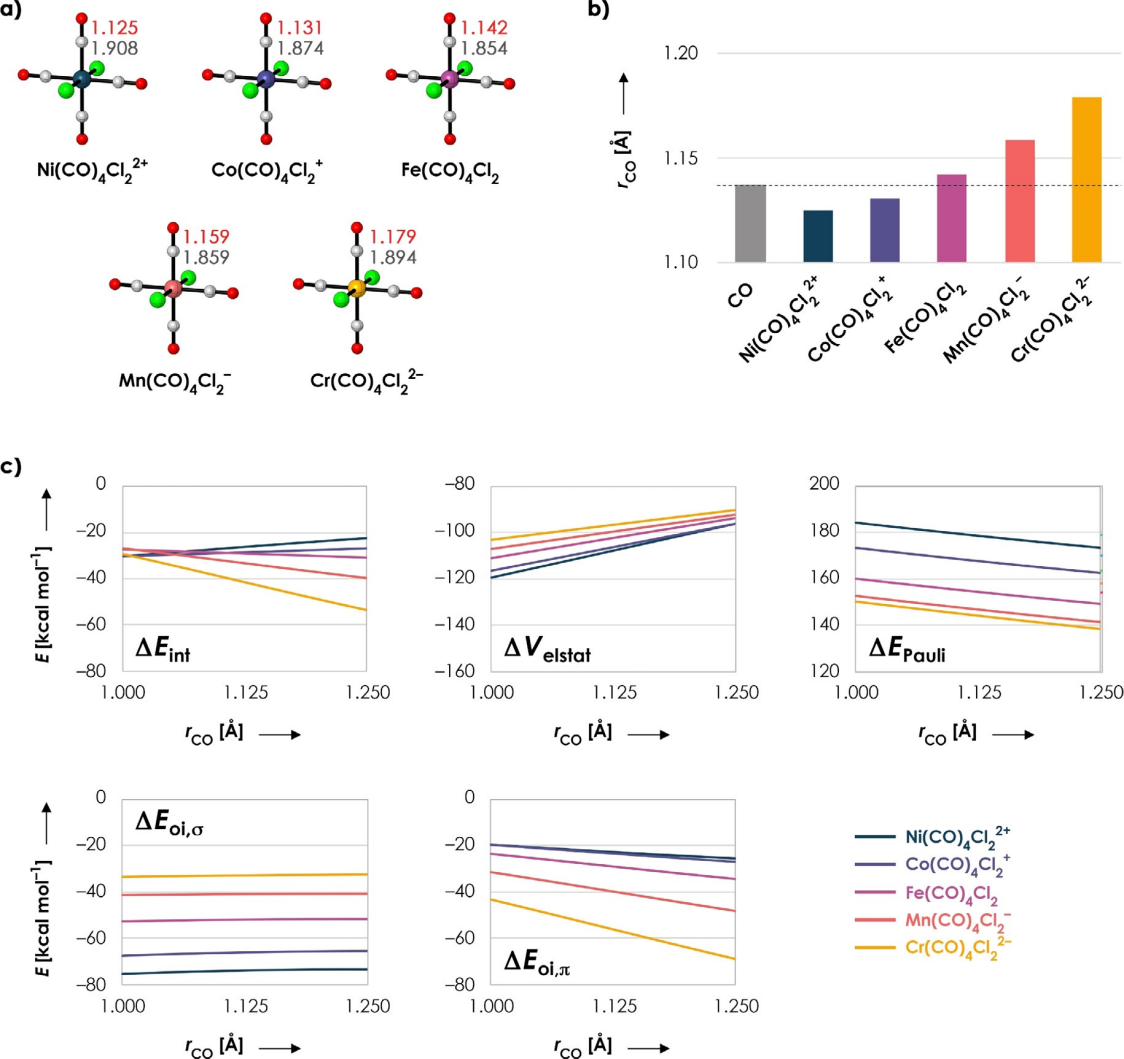
a) Molecular systems with C−O (red) and M−C (gray) distances [in Å]. b) C−O distances [in Å]. The dashed line represents the C−O distance of isolated CO. c) Energy decomposition terms (in kcal mol^−1^) as a function of the C−O distance *r* (in Å) for Ni(CO)_4_Cl_2_^2+^ (dark blue), Co(CO)_4_Cl_2_^+^ (purple), Fe(CO)_4_Cl_2_ (pink), Mn(CO)_4_Cl_2_^−^ (orange) and Cr(CO)_4_Cl_2_^2−^ (yellow). One C−O distance (frag‐CO) has been varied in a stepwise manner from 1.00 to 1.25 Å while keeping its corresponding M−C distance fixed at 1.85 Å; the rest of the system (frag‐M(CO)_3_Cl_2_) is frozen in the same geometry as the fully optimized overall system. All data obtained at the ZORA‐BLYP/TZ2P level of theory.

Finally, we have verified the generality of our results by taking a different set of isoelectronic systems, namely Ni(CO)_4_Cl_2_^2+^, Co(CO)_4_Cl_2_^+^, Fe(CO)_4_Cl_2_, Mn(CO)_4_Cl_2_^−^, and Cr(CO)_4_Cl_2_^2−^ (Figure 8 a). Since these molecules now have two chlorines instead of carbonyl ligands, their chemical properties are different from the systems studied earlier in this work. Nevertheless, we find the same trends as for the systems studied in this manuscript (Figure 8 b), and, more importantly, we find again that the geometrical change of the C−O bond is completely determined by the electrostatic interaction (favoring C−O contraction) and the π‐back‐donation (favoring C−O expansion) (Figure 8 c). A more in‐depth discussion of this dataset is given in Supporting Discussion 2. Hence, we reach identical conclusions with a completely different molecular set, which supports the generality of our findings.

## Conclusions

Whether the C−O bond in carbonyl complexes expands (classical) or contracts (non‐classical) relative to uncoordinated CO is determined by the interplay between electrostatic and π‐back‐bonding orbital interactions in the M−CO coordination bond. The electrostatic M−CO interaction always favors C−O contraction and becomes more pronounced as the effective nuclear charge of the transition metal increases. On the other hand, π‐back‐donation always induces C−O expansion and becomes more important as the complex has a less positive or more negative net charge. This follows from our quantum chemical bonding analyses of five isoelectronic octahedral complexes, namely Fe(CO)_6_^2+^, Mn(CO)_6_^+^, Cr(CO)_6_, V(CO)_6_^−^ and Ti(CO)_6_^2−^, based on relativistic density functional theory at the ZORA‐BLYP/TZ2P level of theory. We have also used a different set of isoelectronic model systems, namely Ni(CO)_4_Cl_2_^2+^, Co(CO)_4_Cl_2_^+^, Fe(CO)_4_Cl_2_, Mn(CO)_4_Cl_2_^−^, and Cr(CO)_4_Cl_2_^2−^, which led to identical conclusions and hence supports the generality of our findings.

The electrostatic M−CO interaction favors C−O contraction for two reasons. The first one is that C−O contraction decreases the distance between the electrons in CO and the nuclei in the rest of the complex, resulting in a stronger electron‐nucleus attraction Δ*V*_e‐N_. As Fe(CO)_6_^2+^ has shorter C−O and C−M distances and, more importantly, the highest nuclear charge, this effect is the most important for Fe(CO)_6_^2+^. The second reason is that C−O contraction decreases the distance between the nuclei in CO and the electrons in the rest of the complex, resulting in a stronger nucleus‐electron attraction Δ*V*_N‐e_. As Fe(CO)_6_^2+^ has the most compact density, which follows again from its high nuclear charge, this effect is the most important for Fe(CO)_6_^2+^. Thus, because of the high nuclear charge of iron, Fe(CO)_6_^2+^ has the strongest tendency for C−O contraction in our series of model systems.

π‐Back‐donation favors C−O expansion because this expansion lowers the energy of the π*‐LUMO on CO, leading to a smaller π‐HOMO–LUMO gap which goes with a more stabilizing orbital interaction. This effect is the most important for Ti(CO)_6_^2−^ because the relatively high 3d orbital in the doubly negatively charged species has the strongest π‐back‐donation. Without any π‐back‐donation, all carbonyl complexes in this study would become nonclassical. Hence, it is the interplay between the electrostatic interaction on one hand (favoring C−O contraction) and π‐back‐donation on the other hand (favoring C−O expansion) that determines the behavior of the CO ligands. The σ‐orbital interaction remains approximately constant when the C−O distance is increased from 1.00 to 1.25 Å, and is therefore not a driving factor that determines whether the system is classical or nonclassical.

## Theoretical Methods

### Computational details

All calculations were performed with the Amsterdam Density Functional (ADF) program 2017.208 at the ZORA‐BLYP/TZ2P level of density functional theory (DFT) for geometry optimizations and energies.[^42^, ^43^, ^44^, ^45^, ^46^, ^47^, ^48^] Geometries were optimized in vacuo with *O_h_* symmetry constraints, and have been verified to be true minima through vibrational analysis (zero imaginary frequencies). The obtained results were verified at two different levels of theory, namely ZORA‐BP86/TZ2P and ZORA‐BLYP‐D3(BJ)/TZ2P, which gave identical trends (Figures S11 and S12). Full computational details are given in Method 1 in the Supporting Information.

### Energy decomposition analysis

The interaction energy between one CO ligand (frag‐CO) and the rest of the complex (frag‐M(CO)_5_) was examined in the framework of Kohn–Sham molecular orbital theory using the quantitative energy decomposition analysis (EDA) scheme.^[39]^ In this fragment‐based approach [Eq. 3], the interaction energy Δ*E*_int_ is decomposed into three physically meaningful and chemically intuitive terms, namely the electrostatic interaction Δ*V*_elstat_, Pauli repulsion Δ*E*_Pauli_ and orbital interactions Δ*E*_oi_:

(3) $$\Delta E{}_{\mathrm{int}}=\Delta V{}_{\mathrm{elstat}}\;+\;\Delta E{}_{\mathrm{Pauli}}\;+\;\Delta E{}_{\mathrm{oi}}$$

The term Δ*V*_elstat_ corresponds to the classical electrostatic interactions between the fragments’ unperturbed charge distributions, and is usually attractive. The Pauli repulsion Δ*E*_Pauli_ comprises the destabilizing interactions between overlapping, occupied orbitals and is responsible for any steric repulsion. The orbital interaction Δ*E*_oi_ accounts for charge transfer (i.e., donor–acceptor interactions between occupied orbitals on one fragment and unoccupied orbitals on the other fragment) and polarization (empty‐occupied orbital mixing on one fragment due to the presence of the other fragment). A theoretical overview of this energy decomposition scheme is given in ref. [39], whereas a step‐by‐step protocol on how to use and interpret the energy decomposition analysis is given in ref. [40].

The orbital interaction energy can be further decomposed into the contributions from each irreducible representation Γ of the point group of the corresponding system. For all fragment analyses, we used *C*_4*v*_ symmetry, which allowed us to decompose Δ*E*_oi_ into a σ and π contribution [Eq. 4]:

(4) $$\Delta E{}_{\mathrm{oi}}=\Delta E{}_{\sigma }\;+\;\Delta E{}_{\pi }$$

## Conflict of interest

The authors declare no conflict of interest.

## Supporting information

As a service to our authors and readers, this journal provides supporting information supplied by the authors. Such materials are peer reviewed and may be re‐organized for online delivery, but are not copy‐edited or typeset. Technical support issues arising from supporting information (other than missing files) should be addressed to the authors.

Supplementary
