# Supplementary material for: The Nature of Nonclassical Carbonyl Ligands Explained by Kohn–Sham Molecular Orbital Theory
Source: Chemistry. 2020 Nov 3;26(67):15690–9. doi: 10.1002/chem.202003768 (PMC7756819; doi:10.1002/chem.202003768)
Supplement: Supplementary file 1 — Supplementary [file CHEM-26-15690-s001.pdf]

Chemistry–A European Journal

Supporting Information

The Nature of Nonclassical Carbonyl Ligands Explained by Kohn–Sham Molecular Orbital Theory

Stephanie C. C. van der Lubbe,[a] Pascal Vermeeren,[a] Célia Fonseca Guerra,[a, b] and F. Matthias Bickelhaupt*[a, c]

Content

Supporting Method 1 | Full computational details S2

Supporting Figure 1 | Energy decomposition analysis graphs with $r_{C\text{-}M}$ = 1.90 Å S3

Supporting Figure 2 | Energy decomposition analysis graphs with $r_{C\text{-}M}$ = 2.00 Å S4

Supporting Figure 3 | Energy decomposition analysis graphs with $r_{C\text{-}M}$ = 2.10 Å S5

Supporting Figure 4 | Isosurfaces of MO 5σ for CO at 1.00 and 1.25 Å S6

Supporting Figure 5 | $Fe(CO)_6^{2+}$ and $Ti(CO)_6^{2-}$ frozen in each other's geometry S7

Supporting Figure 6 | π-orbital diagram CO S8

Supporting Figure 7 | π-HOMO–LUMO overlap as function of r_{CO} S9

Supporting Figure 8 | π-LUMO CO isosurfaces S10

Supporting Figure 9 | Orbital interaction diagrams for constrained systems S11

Supporting Figure 10 | π-HOMO $M(CO)_5$ energies S12

Supporting Figure 11 | Different functionals equilibrium systems S13

Supporting Figure 12 | Different functionals constrained systems S14

Supporting Discussion 1 | The behavior of the Pauli repulsion explained S15

Supporting Discussion 2 | $M(CO)_4Cl_2$ (M=Ni^{2+}, Co^{+}, Fe, Mn^{-}, Cr^{2-}) dataset S17

Supporting Data | Cartesian coordinates S19

Supporting References S21

Supporting Method 1 | Full computational details

All calculations were performed using the Density Functional Theory (DFT) based program Amsterdam Density Functional (ADF) 2017.208.[1-3] We used the BLYP Generalized Gradient Approximation (GGA) density functional, which is composed of the Becke[4] (B) exchange and Lee, Yang and Parr[5] (LYP) correlation functional. Scalar relativistic effects were accounted for by using the zeroth-order regular approximation (ZORA).[6,7] All integrals that are evaluated numerically, including the exchange-correlation integrals, were solved by using the Becke integration scheme with an integration accuracy of 'excellent'.[8]

The Kohn-Sham Molecular Orbitals (KS MOs) were constructed from a linear combination of Slater-type orbitals (STOs), which have the correct cusp behavior and long-range decay. We used the TZ2P basis set, which is of triple-ζ quality for all atoms and has been augmented with two sets of polarization functions, *i.e.*, 3*d* and 4*f* on C and O and 4*p* and 4*f* on Ti, V, Cr, Mn and Fe. No frozen core approximation was used. The molecular density was fitted by the systematically improvable Zlm fitting scheme with quality 'excellent'[9] for all computations except the decomposition of the electrostatic interaction energy term (Equation 1 in manuscript), which was done by using the STO fitting scheme[10] for compatibility reasons. The SCF procedure was considered to be converged if the difference between ρ^{n} and ρ^{n+1} was equal to or smaller than 1e-6.

Geometries were optimized in vacuo in Cartesian coordinates. The convergence criteria were 1e-6 for the changes in bond energy in Hartree, and 1e-5 for the nuclear gradient in Hartree/Ångström. All complexes were optimized with O_h symmetry constraints. The vibrational frequencies were obtained by evaluating the analytical second derivative of the total energy with respect to the nuclear displacements.[11] All optimized structures have been verified to be true minima (zero imaginary frequencies).

Supporting Figure 1 | Energy decomposition analysis graphs with $r_{C\text{-}M}$ = 1.90 Å

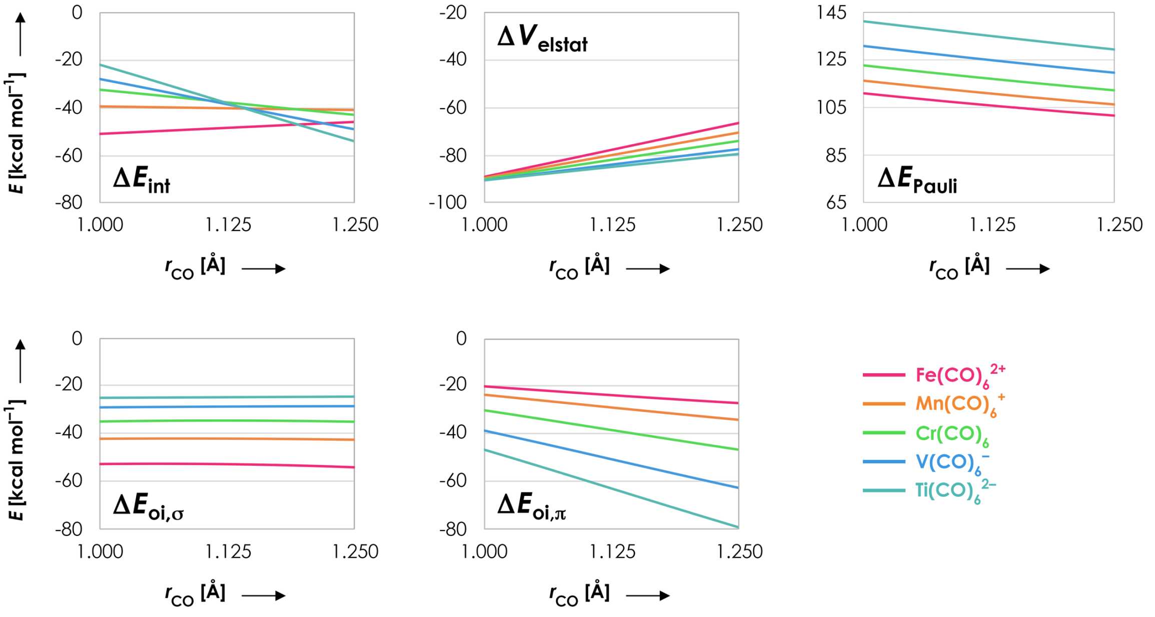

Supporting Figure 1 | Decomposed energy terms (in kcal mol^{-1}) as a function of the C–O distance *r* (in Å) for $Fe(CO)_6^{2+}$ (pink), $Mn(CO)_6^{+}$ (orange), $Cr(CO)_6$ (green), $V(CO)_6^{-}$ (blue) and $Ti(CO)_6^{2-}$ (turquoise). One C–O distance (frag-CO) has been varied in a stepwise manner from 1.00 to 1.25 Å while keeping its corresponding M–C distance fixed at 1.90 Å; the rest of the system (frag-$M(CO)_5$) is frozen in the same geometry as the fully optimized overall system. All data obtained at the ZORA-BLYP/TZ2P level of theory.

Supporting Figure 2 | Energy decomposition analysis graphs with $r_{\text{C-M}}$ = 2.00 Å

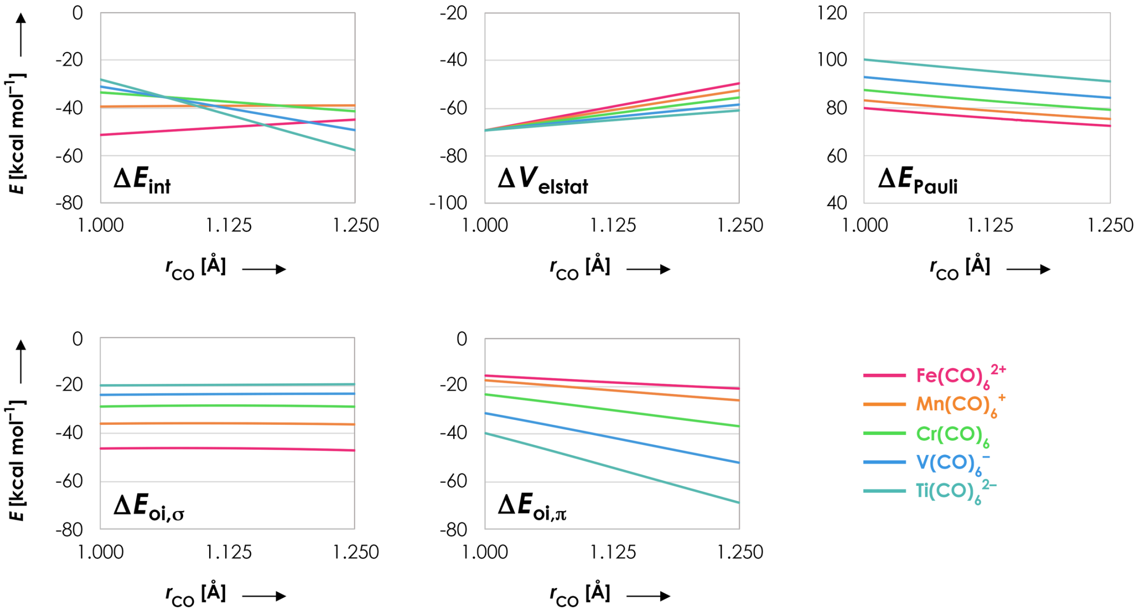

Supporting Figure 2 | Decomposed energy terms (in kcal mol^{-1}) as a function of the C–O distance *r* (in Å) for $Fe(CO)_6^{2+}$ (pink), $Mn(CO)_6^{+}$ (orange), $Cr(CO)_6$ (green), $V(CO)_6^{-}$ (blue) and $Ti(CO)_6^{2-}$ (turquoise). One C–O distance (frag-CO) has been varied in a stepwise manner from 1.00 to 1.25 Å while keeping its corresponding M–C distance fixed at 2.00 Å; the rest of the system (frag-$M(CO)_5$) is frozen in the same geometry as the fully optimized overall system. All data obtained at the ZORA-BLYP/TZ2P level of theory.

Supporting Figure 3 | Energy decomposition analysis graphs with $r_{C\text{-}M}$ = 2.10 Å

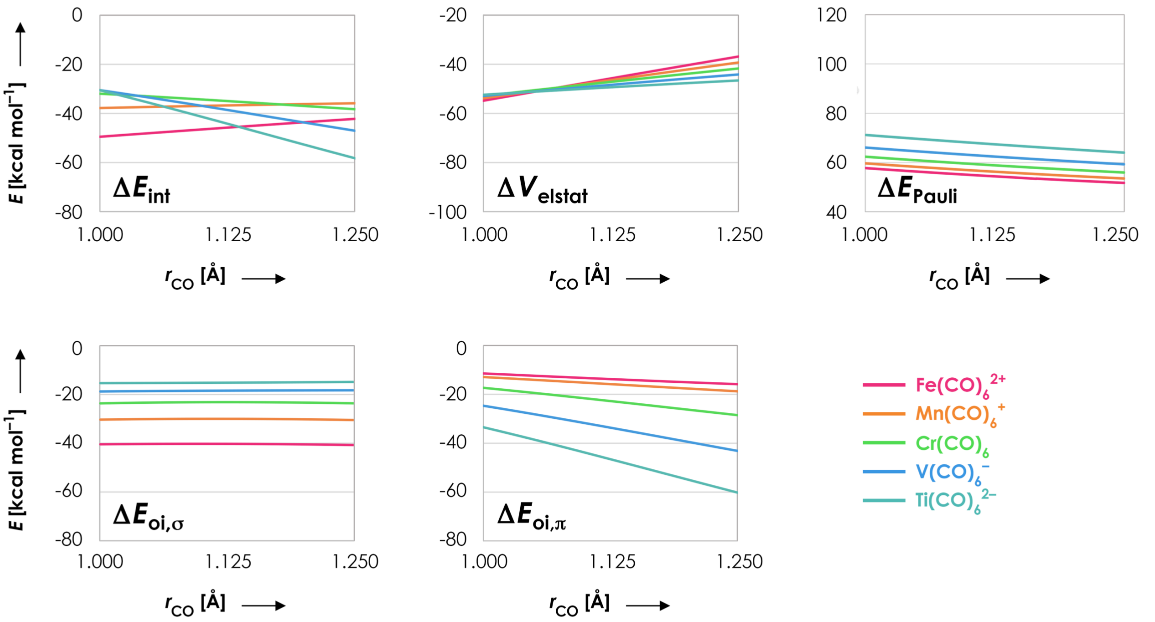

Supporting Figure 3 | Decomposed energy terms (in kcal mol^{-1}) as a function of the C–O distance *r* (in Å) for $Fe(CO)_6^{2+}$ (pink), $Mn(CO)_6^{+}$ (orange), $Cr(CO)_6$ (green), $V(CO)_6^{-}$ (blue) and $Ti(CO)_6^{2-}$ (turquoise). One C–O distance (frag-CO) has been varied in a stepwise manner from 1.00 to 1.25 Å while keeping its corresponding M–C distance fixed at 2.10 Å; the rest of the system (frag-M(CO)$_5$) is frozen in the same geometry as the fully optimized overall system. All data obtained at the ZORA-BLYP/TZ2P level of theory.

Supporting Figure 4 | Isosurfaces of MO 5σ for CO at 1.00 and 1.25 Å

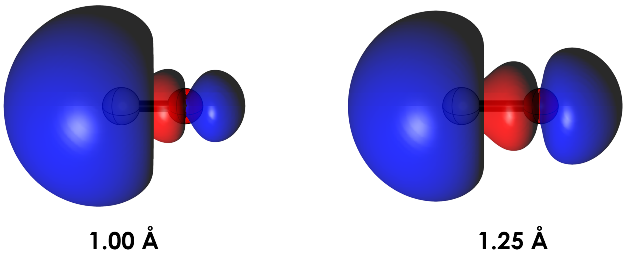

Supporting Figure 4 | Isosurfaces (at 0.03 Bohr$^{-3/2}$) of the 5σ MO for CO at 1.00 Å (left) and 1.25 Å (right), obtained at the ZORA-BLYP/TZ2P level of theory.

Supporting Figure 5 | $Fe(CO)_6^{2+}$ and $Ti(CO)_6^{2-}$ frozen in each other's geometry

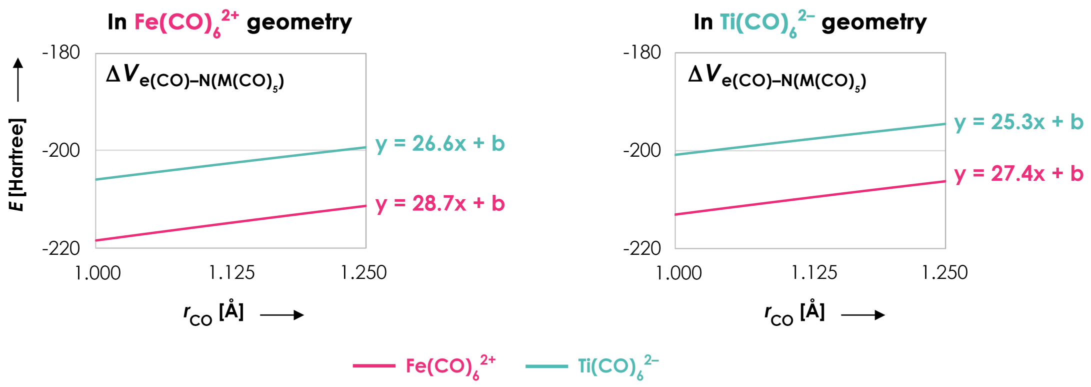

Supporting Figure 5 | Electrostatic interaction [in Hartree] between the electrons in frag-CO and the nuclei in frag-$M(CO)_5$ as a function of the C–O distance *r* (in Å), where M=Fe^{2+} (pink) and Ti^{2-} (turquoise). The make the electron-nucleus distances equal in both systems, $Ti(CO)_5^{2-}$ was frozen in the geometry of $Fe(CO)_5^{2+}$ (left), and $Fe(CO)_5^{2+}$ was frozen in the geometry of $Ti(CO)_5^{2-}$ (right); the C–O distance of frag-CO has been varied in a stepwise manner from 1.00 to 1.25 Å while keeping its corresponding M–C distance fixed at 1.95 Å. The linear equations are given to see the differences in slope (R^2=1.00 for each linear regression), which goes from 3.4 in their equilibrium geometries to 2.1 when both systems share the same geometry. Hence, even though the positions of the nuclei are now the same for both systems, $Fe(CO)_6^{2+}$ still has a larger tendency for C–O contraction than $Ti(CO)_6^{2-}$. All data obtained at the ZORA-BLYP/TZ2P level of theory.

Supporting Figure 6 | π-orbital diagram CO

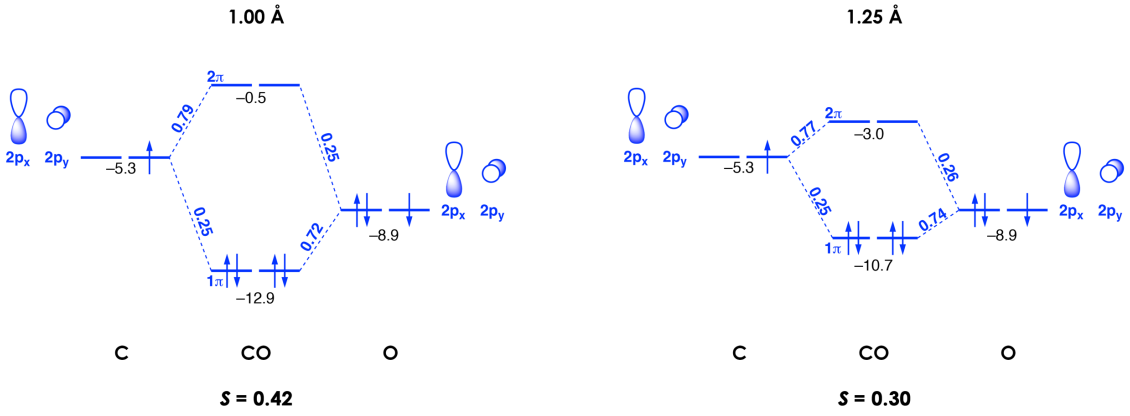

Supporting Figure 6 | π molecular orbital diagram for CO at *r* = 1.00 Å (left) and *r* = 1.25 Å (right) with orbital energies [in eV] in black and SFO contributions in blue. As the C–O distance decreases, the overlap between the atomic orbitals increases from 0.30 (1.25 Å) to 0.42 (1.00 Å), which stabilizes the bonding orbital 1π and destabilizes the antibonding orbital 2π. All data obtained at the ZORA-BLYP/TZ2P level of theory.

Supporting Figure 7 | π-HOMO–LUMO overlap as function of r_{CO}

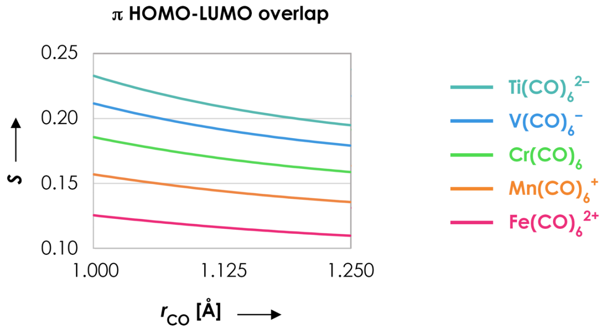

Supporting Figure 7 | The π-HOMO–LUMO overlap as a function of the C–O distance *r* (in Å) for $Fe(CO)_6^{2+}$ (pink), $Mn(CO)_6^{+}$ (orange), $Cr(CO)_6$ (green), $V(CO)_6^{-}$ (blue) and $Ti(CO)_6^{2-}$ (turquoise). One C–O distance (frag-CO) has been varied in a stepwise manner from 1.00 to 1.25 Å while keeping its corresponding M–C distance fixed at 1.95 Å; the rest of the system (frag-$M(CO)_5$) is frozen in the same geometry as the fully optimized overall system. For each system, the overlap increases upon decreasing the C–O distance. The overlap changes the fastest for $Ti(CO)_6^{2-}$ (largest slope) and slowest for $Fe(CO)_6^{2+}$ (smallest slope). As a larger overlap gives stronger orbital interactions, the π-HOMO–LUMO overlap is not responsible for the weakening of $\Delta E_{oi,\pi}$ upon CO compression, and furthermore does not explain why this weakening is the strongest for $Ti(CO)_6^{2-}$. All data obtained at the ZORA-BLYP/TZ2P level of theory.

Supporting Figure 8 | π-LUMO CO isosurfaces

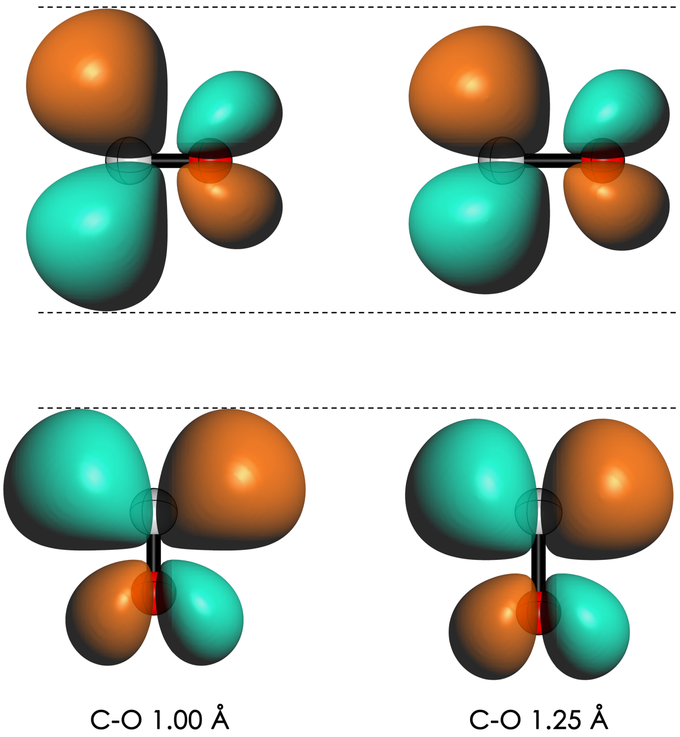

Supporting Figure 8 | Isosurfaces (at 0.05 $Bohr^{-3/2}$) of the π-LUMO of CO at 1.00 Å (left) and 1.25 Å (right). The dashed lines serve to make a visual comparison more straightforward. The amplitude on the C atom becomes larger at shorter C–O distance, which explains the increase in overlap with the π-HOMO on frag-$M(CO)_5$ when the C–O distance is decreased. Data obtained at the ZORA-BLYP/TZ2P level of theory.

Supporting Figure 9 | Orbital interaction diagrams for constrained systems

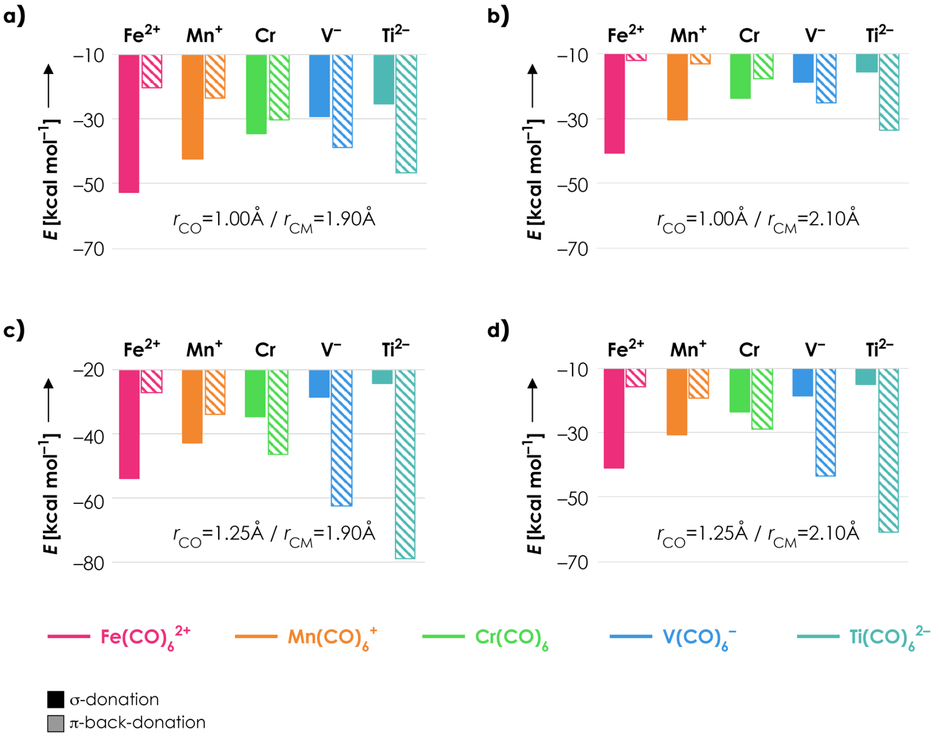

Supporting Figure 9 | σ– (solid) and π– (striped) orbital interactions (in kcal mol^{-1}) between C–O and $Fe(CO)_5^{2+}$ (pink), $Mn(CO)_5^{+}$ (orange), $Cr(CO)_5$ (green), $V(CO)_5^{-}$ (blue) and $Ti(CO)_5^{2-}$ (turquoise). The C-O distance of frag-CO and M–C distance between frag-CO and frag-M(CO)$_5$ has been constrained at **a)** 1.00 and 1.90 Å, **b)** 1.00 and 2.10 Å, **c)** 1.25 and 1.90 Å and **d)** 1.25 and 2.10 Å, respectively, while the rest of the system (M(CO)$_5$) has been frozen in the same geometry as the fully optimized overall system. All data obtained at the ZORA-BLYP/TZ2P level of theory.

Supporting Figure 10 | π-HOMO $M(CO)_5$ energies

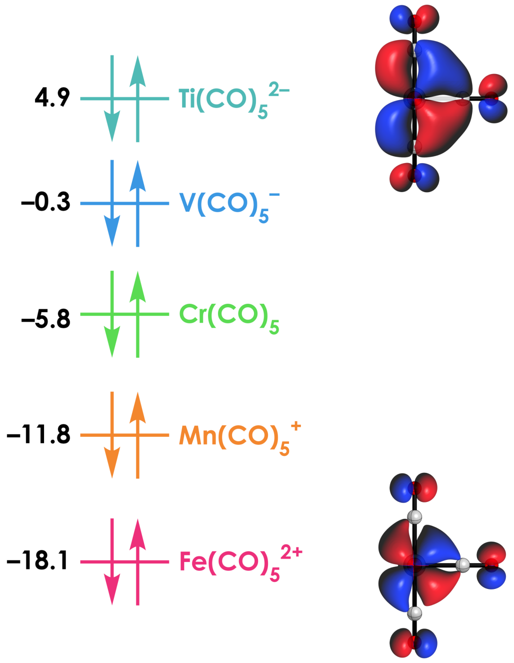

Supporting Figure 10 | Energies of the π-HOMO (in eV) for $Fe(CO)_5^{2+}$ (pink), $Mn(CO)_5^{+}$ (orange), $Cr(CO)_5$ (green), $V(CO)_5^{-}$ (blue) and $Ti(CO)_5^{2-}$ (turquoise) in the geometry of the fully optimized overall system and the corresponding isosurfaces (at 0.03 Bohr$^{-3/2}$) for $Fe(CO)_5^{2+}$ (down) and $Ti(CO)_5^{2-}$ (up). Data obtained at the ZORA-BLYP/TZ2P level of theory.

Supporting Figure 11 | Different functional equilibrium systems

ZORA-BLYP-D3(BJ)/TZ2P

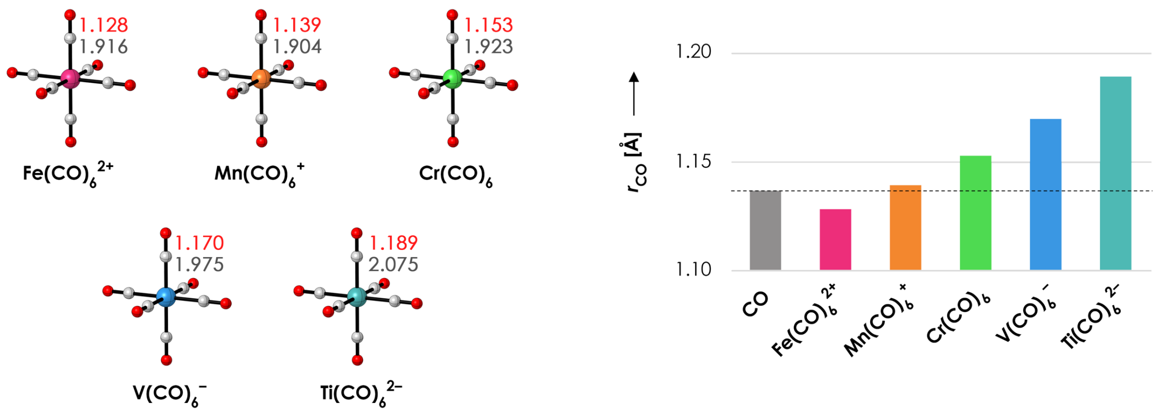

ZORA-BP86/TZ2P

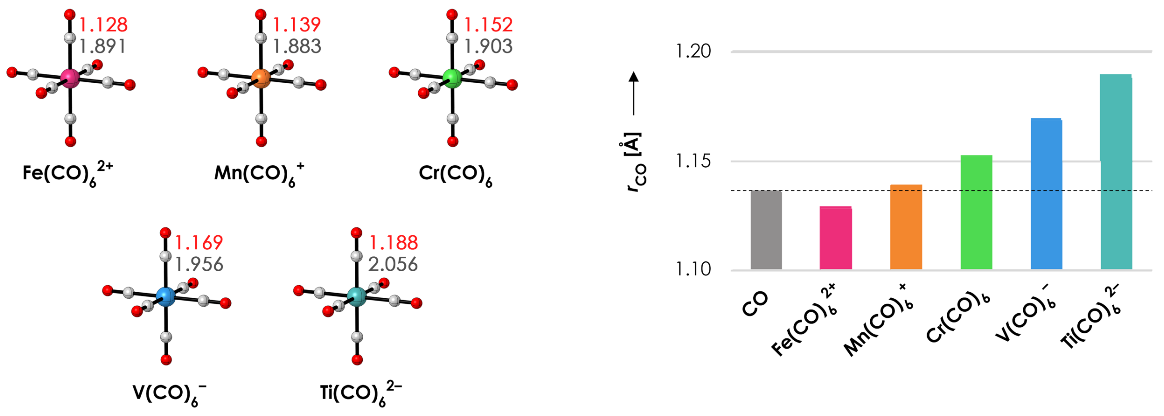

Supporting Figure 11 | Studied molecular systems with C–O (red) and M–C (gray) distances [in Å] (left), and the C–O distances [in Å] in comparison with the C–O distance of isolated CO (dashed line) (right), obtained at the ZORA-BLYP-D3(BJ)/TZ2P level of theory (top) and ZORA-BP86/TZ2P level of theory (bottom). These different levels of theory give identical trends as the ZORA-BLYP/TZ2P level of theory that was used in our work.

Supporting Figure 12 | Different functional constrained systems

ZORA-BLYP-D3(BJ)/TZ2P

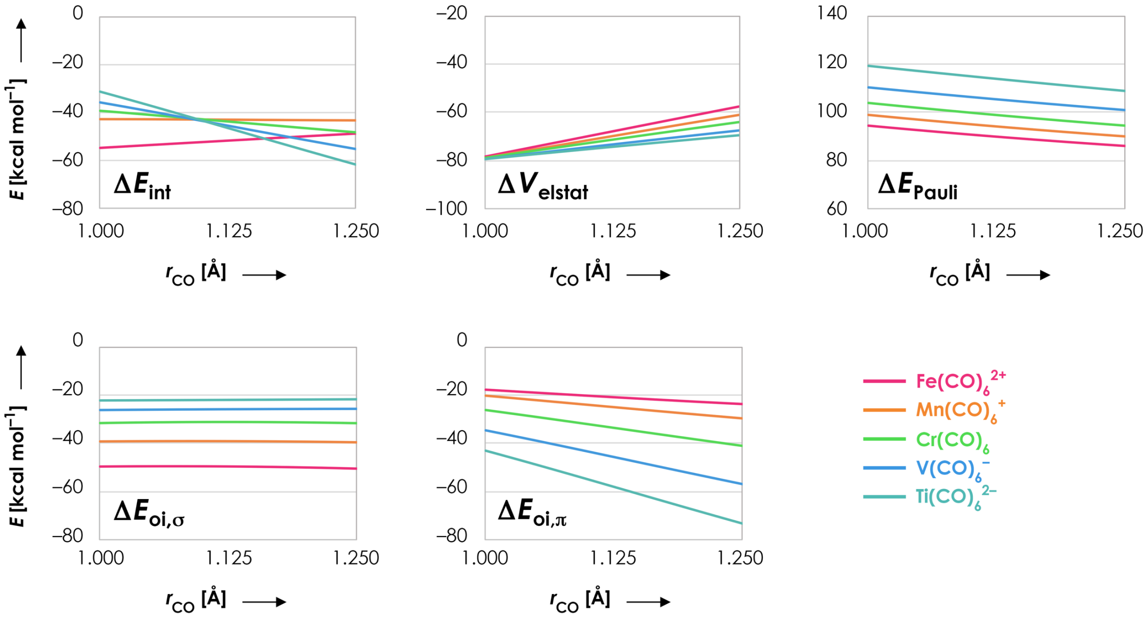

ZORA-BP86/TZ2P

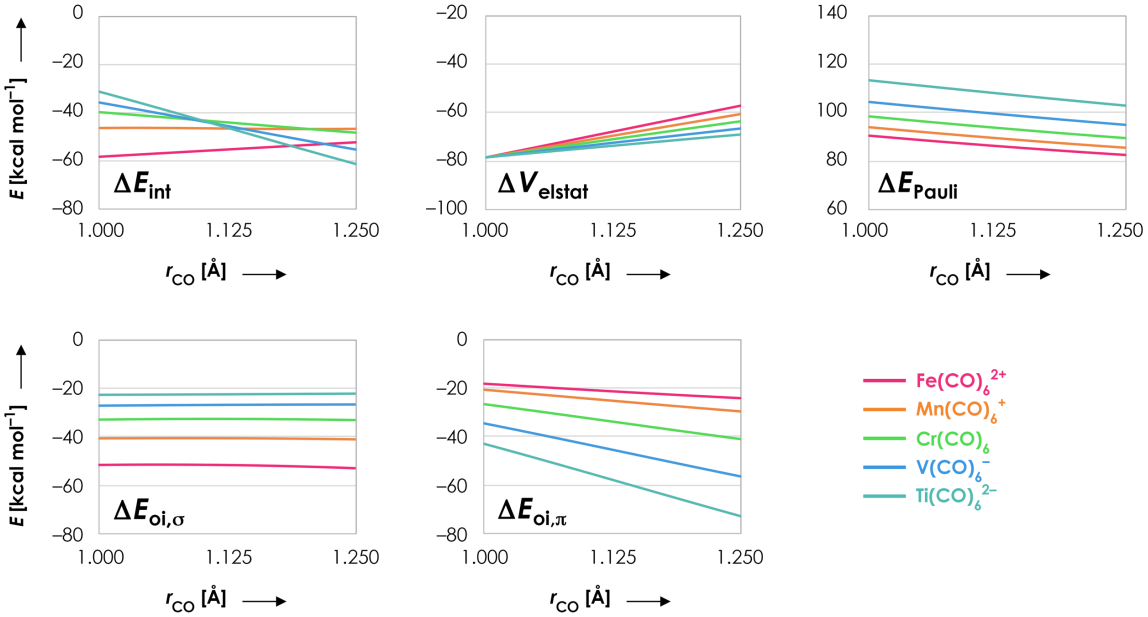

Supporting Figure 12 | Energy decomposition terms (in kcal mol^{-1}) as a function of the C–O distance *r* (in Å) for $Fe(CO)_6^{2+}$ (pink), $Mn(CO)_6^{+}$ (orange), $Cr(CO)_6$ (green), $V(CO)_6^{-}$ (blue) and $Ti(CO)_6^{2-}$ (turquoise). One C–O distance (frag-CO) has been varied in a stepwise manner from 1.00 to 1.25 Å while keeping its corresponding C–M distance fixed at 1.95 Å; the rest of the system (frag-M$(CO)_5$) is frozen in the same geometry as the fully optimized overall system. Data obtained at the ZORA-BLYP-D3(BJ)/TZ2P level of theory (top) and ZORA-BP86/TZ2P level of theory (bottom). These different levels of theory give identical trends as the ZORA-BLYP/TZ2P level of theory that was used in our work.

Supporting Discussion 1 | The behavior of the Pauli repulsion explained

The Pauli repulsion becomes stronger when CO is contracted for all five systems (Figure 2 in manuscript), and increases in strength when going from $Fe(CO)_6^{2+}$ to $Ti(CO)_6^{2-}$. Both observations can be understood by analyzing the overlap between the filled fragment molecular orbitals (FMOs).

We start by rationalizing the differences in Pauli repulsion between the different systems. As can be seen in Supporting Figure 13a, the overlap between the filled FMOs is the smallest for $Fe(CO)_6^{2+}$ and largest for $Ti(CO)_6^{2-}$, which explains why $Fe(CO)_6^{2+}$ has the weakest while $Ti(CO)_6^{2-}$ has the strongest Pauli repulsion. The reason why the overlap is largest for $Ti(CO)_6^{2-}$ is because the electronic density in $Ti(CO)_5^{2-}$ is more diffuse than the electronic density in $Fe(CO)_5^{2+}$ (see also Figure 6 in manuscript), resulting in a larger overlap with the FMOs of CO.

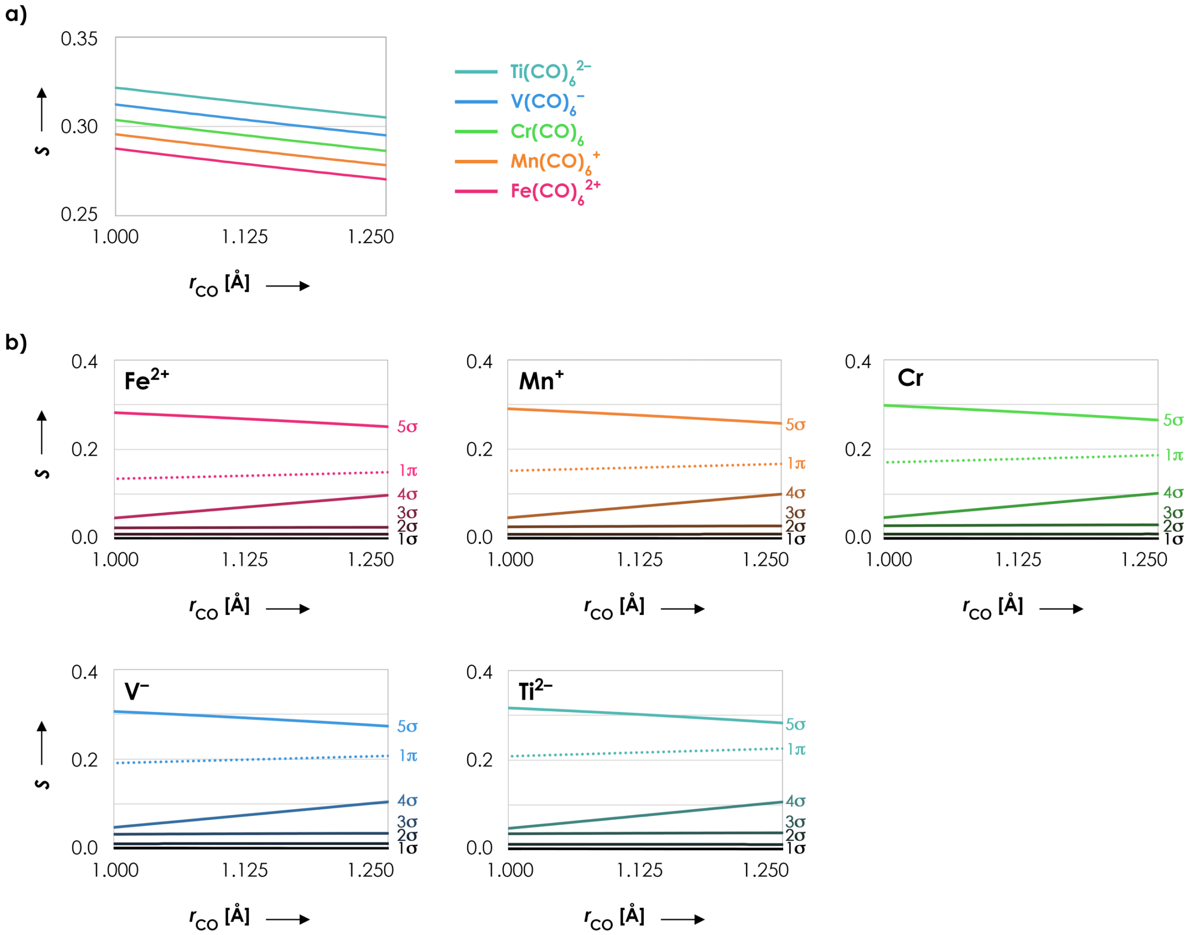

Supporting Figure 13 | Orbital overlap as a function of the C–O distance *r* (in Å) for $Fe(CO)_6^{2+}$ (pink), $Mn(CO)_6^{+}$ (orange), $Cr(CO)_6$ (green), $V(CO)_6^{-}$ (blue) and $Ti(CO)_6^{2-}$ (turquoise). One C–O distance (frag-CO) has been varied in a stepwise manner from 1.00 to 1.25 Å while keeping its corresponding M–C distance fixed at 1.95 Å; the rest of the system (frag-$M(CO)_5$) is frozen in the same geometry as the fully optimized overall system. **a)** Square root over the squared sum of all possible overlaps between the fragment's filled molecular orbitals. **b)** Square root over the squared sum of the overlaps between one filled FMO in CO with all filled FMOs in frag-$M(CO)_5$. All data obtained at the ZORA-BLYP/TZ2P level of theory.

Next, we explain why the Pauli repulsion increases when the C–O bond length decreases. Supporting Figure 13b shows the overlap for each filled FMO on CO with all filled FMOs on frag-$M(CO)_5$. From this figure, it becomes clear that the destabilization with decreased C–O distance is completely caused by the overlap between the 5σ orbital on CO with the filled FMOs on frag-$M(CO)_5$. After all, this is the only line with a negative slope, while all other lines are either flat or have a positive slope. The reason that decreasing the C–O distance leads to a larger overlap between the CO 5σ and the filled FMOs on frag-$M(CO)_5$ is that C–O contraction results in a larger amplitude on the carbon atom (see also Supporting Figure 4). Hence, the Pauli repulsion increases with decreasing C–O distance because C–O contraction results in a 5σ orbital with a larger lobe on the carbon atom. This larger lobe results in a larger overlap with the filled FMOs on frag-$M(CO)_5$, and thus a larger Pauli repulsion.

Supporting Discussion 2 | $M(CO)_4Cl_2$ ($M=Ni^{2+}$, Co^+, Fe, Mn^-, Cr^{2-}) dataset

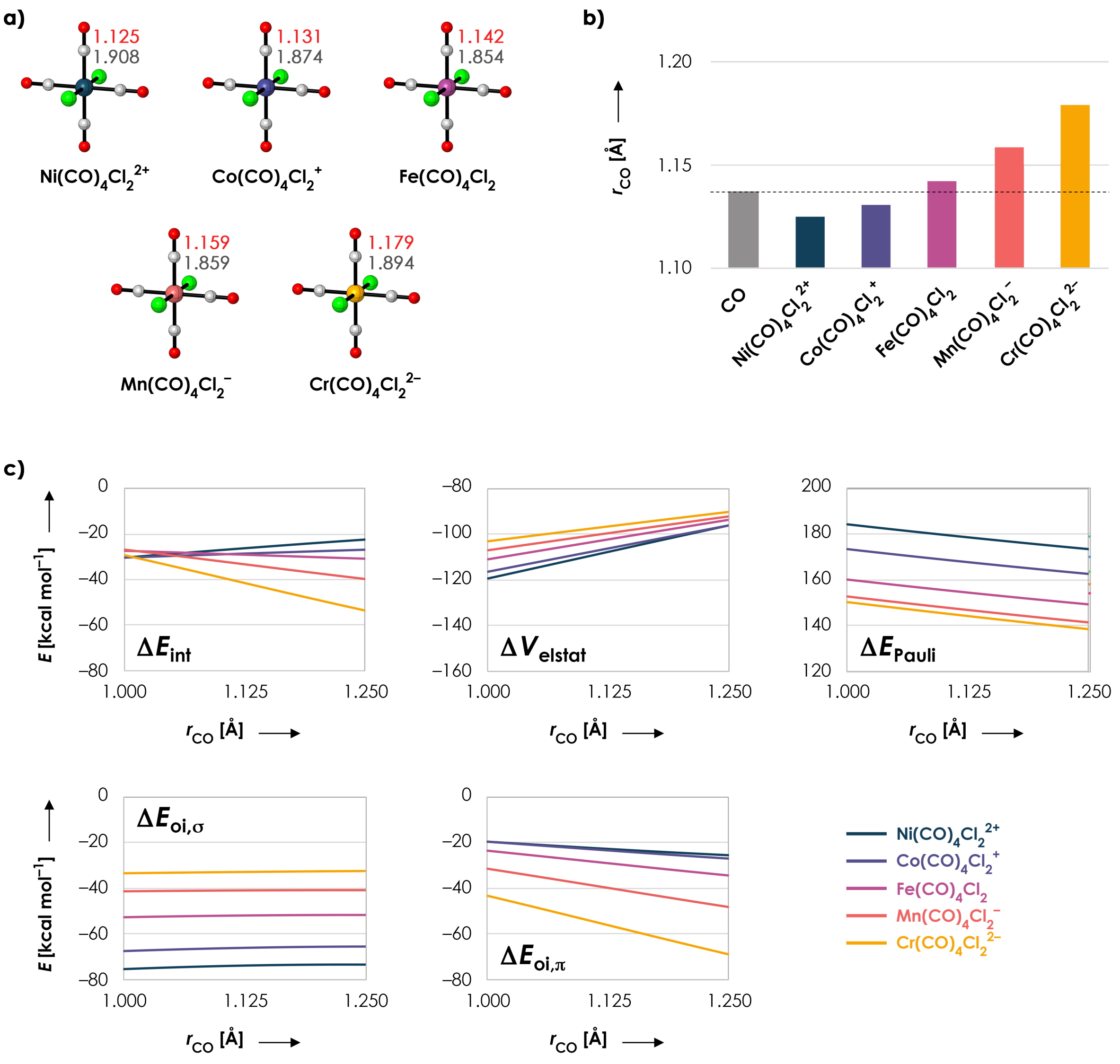

Supporting Figure 14 | a) Molecular systems with C–O (red) and M–C (gray) distances [in Å]. **b)** C–O distances [in Å]. The dashed line represents the C–O distance of isolated CO. **c)** Energy decomposition terms (in kcal mol^{-1}) as a function of the C–O distance r (in Å) for $Ni(CO)_4Cl_2^{2+}$ (dark blue), $Co(CO)_4Cl_2^{+}$ (purple), $Fe(CO)_4Cl_2$ (pink), $Mn(CO)_4Cl_2^{-}$ (orange) and $Cr(CO)_4Cl_2^{2-}$ (yellow). One C–O distance (frag-CO) has been varied in a stepwise manner from 1.00 to 1.25 Å while keeping its corresponding M–C distance fixed at 1.85 Å; the rest of the system (frag-$M(CO)_3Cl_2$) is frozen in the same geometry as the fully optimized overall system. All data obtained at the ZORA-BLYP/TZ2P level of theory. This figure is the same as Figure 8 in the manuscript, but is given again to improve the readability of this supporting discussion.

To verify the generality of our findings, we studied a different set of isoelectronic systems, namely $Ni(CO)_4Cl_2^{2+}$, $Co(CO)_4Cl_2^{+}$, $Fe(CO)_4Cl_2$, $Mn(CO)_4Cl_2^{-}$, and $Cr(CO)_4Cl_2^{2-}$. These systems have two isomeric forms, the cis conformation (global minima), in which the carbonyl ligands slightly bend in the direction of the chlorides, and trans conformation (local minima), in which all carbonyl ligands are surrounded by the same molecular environment if D_{4h} symmetry is enforced. The $Co(CO)_4Cl_2^{+}$,

$Fe(CO)_4Cl_2$ and $Mn(CO)_4Cl_2^{-}$ trans isomers do indeed have D_{4h} symmetry, but the $Ni(CO)_4Cl_2^{2+}$ and $Cr(CO)_4Cl_2^{2-}$ trans isomers have C_2 and D_{2d} symmetry, respectively. Yet, we studied all systems in trans conformation with D_{4h} symmetry for the following two reasons. Firstly, we are purely interested in the effect of C–O contraction and expansion, so we inhibited the introduction of any secondary effects such as varying degrees of carbonyl-chloride interactions. Secondly, the use of D_{4h} symmetry allows us to decompose the orbital interactions into σ and π contributions, providing us with additional chemical insight.

As can be seen in Supporting Figure 14a, the C–O bond length is the shortest in $Ni(CO)_4Cl_2^{2+}$ (1.125 Å), and gradually expands as the charge goes from +2 to –2, reaching a maximum in $Cr(CO)_4Cl_2^{2-}$ (1.179Å). This gradual increase is in line with the findings in our manuscript. Comparing these bond lengths with the bond length of isolated CO (Supporting Figure 14b), we see that both positively charged systems are nonclassical, while the neutral and negatively charged systems are classical.

In line with these observations is the interaction energy ΔE_{int} as a function of the C–O bond length in Supporting Figure 14c. This figure is obtained in the same way as Figure 2 in the manuscript; the only difference is that the C-M distance is 1.85 instead of 1.95 Å because this distance is closer to the fully optimized C–M bond lengths. $Ni(CO)_4Cl_2^{2+}$ has the strongest tendency for C–O contraction (largest positive slope) while $Cr(CO)_4Cl_2^{2-}$ has the strongest tendency for C–O expansion (largest negative slope). These different tendencies for C–O expansion and contraction are caused by two factors, namely the electrostatic interaction and π-back-donation.

The electrostatic interaction favors C–O contraction for all five systems, but the tendency for C–O contraction is the largest for $Ni(CO)_4Cl_2^{2+}$ (largest positive slope) and smallest for $Cr(CO)_4Cl_2^{2-}$ (smallest positive slope). The π-back-donation favors C–O expansion for all five systems, but the tendency for C–O expansion is the largest for $Cr(CO)_4Cl_2^{2-}$ (largest negative slope) and smallest for $Ni(CO)_4Cl_2^{2+}$ (smallest negative slope). The Pauli repulsion and σ-orbital interaction have the same slope for all five molecular systems and are therefore not responsible for the different tendencies among the different systems. Hence, it is an interplay between the electrostatic interactions (favoring C–O contraction) and π-back-donation (favoring C–O expansion) that determines whether the system will be classical or nonclassical. These conclusions are exactly the same as obtained in the manuscript with the systems $Fe(CO)_6^{2+}$, $Mn(CO)_6^{+}$, $Cr(CO)_6$, $V(CO)_6^{-}$ and $Ti(CO)_6^{2-}$, which supports the generality of our findings.

Cartesian coordinates [in Å] and total bonding energies [in kcal mol^{-1}] of all the fully optimized systems used in this work, computed at ZORA-BLYP/TZ2P level of theory.

CO, 0 imaginary frequencies [-332.6]

O	0.000000	3.079531	0.000000
C	0.000000	1.942795	0.000000

$Fe(CO)_6^{2+}$, 0 imaginary frequencies [-1816.2]

Fe	0.000000	0.000000	0.000000
O	0.000000	3.052965	0.000000
O	0.000000	0.000000	3.052965
O	0.000000	-3.052965	0.000000
O	3.052965	0.000000	0.000000
O	0.000000	0.000000	-3.052965
O	-3.052965	0.000000	0.000000
C	1.924341	0.000000	0.000000
C	0.000000	0.000000	-1.924341
C	0.000000	-1.924341	0.000000
C	-1.924341	0.000000	0.000000
C	0.000000	0.000000	1.924341
C	0.000000	1.924341	0.000000

$Mn(CO)_6^{+}$, 0 imaginary frequencies [-2133.3]

Mn	0.000000	0.000000	0.000000
O	0.000000	3.050664	0.000000
O	0.000000	0.000000	3.050664
O	0.000000	-3.050664	0.000000
O	3.050664	0.000000	0.000000
O	0.000000	0.000000	-3.050664
O	-3.050664	0.000000	0.000000
C	1.911527	0.000000	0.000000
C	0.000000	0.000000	-1.911527
C	0.000000	-1.911527	0.000000
C	-1.911527	0.000000	0.000000
C	0.000000	0.000000	1.911527
C	0.000000	1.911527	0.000000

$Cr(CO)_6$, 0 imaginary frequencies [-2306.1]

Cr	0.000000	0.000000	0.000000
O	0.000000	3.083069	0.000000
O	0.000000	0.000000	3.083069
O	0.000000	-3.083069	0.000000
O	3.083069	0.000000	0.000000
O	0.000000	0.000000	-3.083069
O	-3.083069	0.000000	0.000000
C	1.929725	0.000000	0.000000
C	0.000000	0.000000	-1.929725
C	0.000000	-1.929725	0.000000
C	-1.929725	0.000000	0.000000
C	0.000000	0.000000	1.929725
C	0.000000	1.929725	0.000000

$V(CO)_6^{-}$, 0 imaginary frequencies [-2326.6]

V	0.000000	0.000000	0.000000
O	0.000000	3.153229	0.000000
O	0.000000	0.000000	3.153229
O	0.000000	-3.153229	0.000000
O	3.153229	0.000000	0.000000
O	0.000000	0.000000	-3.153229
O	-3.153229	0.000000	0.000000
C	1.982786	0.000000	0.000000
C	0.000000	0.000000	-1.982786
C	0.000000	-1.982786	0.000000
C	-1.982786	0.000000	0.000000
C	0.000000	0.000000	1.982786
C	0.000000	1.982786	0.000000

$Ti(CO)_6^{2-}$, 0 imaginary frequencies [-2216.0]

Ti	0.000000	0.000000	0.000000
O	0.000000	3.273870	0.000000
O	0.000000	0.000000	3.273870
O	0.000000	-3.273870	0.000000
O	3.273870	0.000000	0.000000
O	0.000000	0.000000	-3.273870
O	-3.273870	0.000000	0.000000
C	2.084356	0.000000	0.000000
C	0.000000	0.000000	-2.084356
C	0.000000	-2.084356	0.000000
C	-2.084356	0.000000	0.000000
C	0.000000	0.000000	2.084356
C	0.000000	2.084356	0.000000

$Ni(CO)_4Cl_2^{2+}$, D_{4h} constrained [-1042.1]

Ni	0.000000	0.000000	0.000000
O	2.144072	-2.144072	0.000000
O	2.144072	2.144072	0.000000
O	-2.144072	2.144072	0.000000
C	1.348867	-1.348867	0.000000
O	-2.144072	-2.144072	0.000000
C	1.348867	1.348867	0.000000
Cl	0.000000	0.000000	2.280426
C	-1.348867	-1.348867	0.000000
C	-1.348867	1.348867	0.000000
Cl	0.000000	0.000000	-2.280426

$Co(CO)_4Cl_2^{+}$, D_{4h} constrained [-1462.5]

Co	0.000000	0.000000	0.000000
O	2.124861	-2.124861	0.000000
O	2.124861	2.124861	0.000000
O	-2.124861	2.124861	0.000000
C	1.325384	-1.325384	0.000000
O	-2.124861	-2.124861	0.000000
C	1.325384	1.325384	0.000000
Cl	0.000000	0.000000	2.283595
C	-1.325384	-1.325384	0.000000
C	-1.325384	1.325384	0.000000
Cl	0.000000	0.000000	-2.283595

$Fe(CO)_4Cl_2$, D_{4h} constrained [-1692.3]

Fe	0.000000	0.000000	0.000000
O	2.118801	-2.118801	0.000000
O	2.118801	2.118801	0.000000
O	-2.118801	2.118801	0.000000
C	1.311236	-1.311236	0.000000
O	-2.118801	-2.118801	0.000000
C	1.311236	1.311236	0.000000
Cl	0.000000	0.000000	2.349233
C	-1.311236	-1.311236	0.000000
C	-1.311236	1.311236	0.000000
Cl	0.000000	0.000000	-2.349233

$Mn(CO)_4Cl_2^{-}$, D_{4h} constrained [-1777.7]

Mn	0.000000	0.000000	0.000000
O	2.133628	-2.133628	0.000000
O	2.133628	2.133628	0.000000
O	-2.133628	2.133628	0.000000
C	1.314427	-1.314427	0.000000
O	-2.133628	-2.133628	0.000000
C	1.314427	1.314427	0.000000
Cl	0.000000	0.000000	2.465749
C	-1.314427	-1.314427	0.000000
C	-1.314427	1.314427	0.000000
Cl	0.000000	0.000000	-2.465749

$Cr(CO)_4Cl_2^{2-}$, D_{4h} constrained [1734.6]

Cr	0.000000	0.000000	0.000000
O	2.172517	2.172517	0.000000
O	2.172517	-2.172517	0.000000
O	-2.172517	-2.172517	0.000000
C	1.338941	1.338941	0.000000
O	-2.172517	2.172517	0.000000
C	1.338941	-1.338941	0.000000
Cl	0.000000	0.000000	-2.677281
C	-1.338941	1.338941	0.000000
C	-1.338941	-1.338941	0.000000
Cl	0.000000	0.000000	2.677281

Supporting References

[1] *ADF2017*, SCM, Theoretical Chemistry, Vrije Universiteit, Amsterdam, The Netherlands, http://www.scm.com.

[2] G. te Velde et al., *J. Comput. Chem.* **2001**, *22*, 931.

[3] C. Fonseca Guerra, J. G. Snijders, G. te Velde, E. J. Baerends, *Theor. Chem. Acc.* **1998**, *99*, 391.

[4] A. D. Becke, *Phys. Rev. A* **1988**, *38*, 3098.

[5] C. Lee, W. Yang, R. G. Parr, *Phys. Rev. B* **1988**, *37*, 785.

[6] E. van Lenthe, R. van Leeuwen, E. J. Baerends, J. G. Snijders, *Int. J. Quantum Chem.* **1996**, *57*, 281.

[7] E. van Lenthe, E. J. Baerends, J. G. Snijders, *J. Chem. Phys.* **1994**, *101*, 9783.

[8] A. D. Becke, *J. Chem. Phys.* **1988**, *88*, 2547.

[9] M. Franchini, P. H. T. Philipsen, E. van Lenthe, L. Visscher, *J. Chem. Theory Comput.* **2014**, *10*, 1994.

[10] E. J. Baerends, D. E. Ellis, P. Ros, *Chem. Phys.* **1973**, *2*, 41.

[11] S. K. Wolff, *Int. J. Quantum Chem.* **2005**, *104*, 645.

[12] F. M. Bickelhaupt, E. J. Baerends in *Reviews in Computational Chemistry*, Vol. 15 (Eds.: K. B. Lipkowitz, D. B. Boyd), Wiley-VCH, New York, **2000**, pp. 1-86.

[13] T. Ziegler, A. Rauk, *Inorg. Chem.* **1979**, *18*, 1755.

[14] T. Ziegler, A. Rauk, *Inorg. Chem.* **1979**, *18*, 1558.

[15] T. Ziegler, A. Rauk, *Theor. Chi. Acta* **1977**, *46*, 1.

[16] K. Morokuma, *J. Chem. Phys.* **1971**, *55*, 1236.

[17] K. Kitaura, K. Morokuma, *Int. J. Quantum Chem.* **1976**, *10*, 325.

[18] F. M. Bickelhaupt, J. K. Nagle, W. L. Klemm, *J. Phys. Chem. A* **2008**, *112*, 2437.
